# Highly Controlled Nanostructured CuO Photocathodes

**DOI:** 10.1021/acsanm.6c01037

**Published:** 2026-06-27

**Authors:** Javier Prieto-Serrano, Miguel García-Tecedor, Mariam Barawi, Miguel Gomez-Mendoza, María Alcaire, Ana Borras, Víctor A. de la Peña O’Shea, José A. Martín-Gago, María F. López, Lidia Martínez

**Affiliations:** † Instituto de Ciencia de Materiales de Madrid (ICMM), CSIC, Madrid 28049, Spain; ‡ IMDEA-Energía, Móstoles 28935, Spain; § Instituto de Ciencia de Materiales de Sevilla (ICMS), CSIC − US, Sevilla 41092, Spain

**Keywords:** CuO nanoparticles, CuO nanocubes, thermal treatments, photoelectrocatalysis, gas aggregation sources

## Abstract

Today, Cu-based photocathodes are increasingly employed due to Cu abundance, environmental benefits, and high photoelectrocatalytic activity. However, despite significant advances in this field, the development of controllable methodologies to produce highly crystalline, structurally tailored, and reproducible CuO nanoarchitectures remains challenging. Here, we propose an alternative route for the fabrication of nanostructured CuO photocathodes that can address these goals. We have grown highly controlled CuO nanoparticles onto fluorine-doped tin oxide, FTO, electrodes by using a sputter gas aggregation source. The photoelectrochemical response was optimized by different thermal treatments, tuning the environment, duration, and temperature. We have systematically studied the influence of the treatments on the structure of the films and their correlation with the photoresponse of the photoelectrodes. We showed that the thermal treatments first induced nanoparticle growth, which is subsequently followed by coalescence. The best photoelectrochemical performance was obtained after complete recrystallization in the form of CuO nanocubes (thermal treatment in vacuum, 18 h, 500 °C), resulting in a porous film photocathode. This electrode presents maximum current densities of −1.2 mA/cm^2^, an applied bias photon-to-current efficiency of 1.4%, and a moderate operational stability for bare CuO photocathodes operating in aqueous electrolyte. The structural changes with improved crystallinity play a crucial role in favoring charge transport and reducing the recombination of photogenerated electron–hole pairs, thereby enhancing the photocurrent generated by the photocathode. Therefore, this methodology for producing CuO nanostructured films emerges as an alternative approach for fabricating photoelectrocathodes.

## Graphical abstract

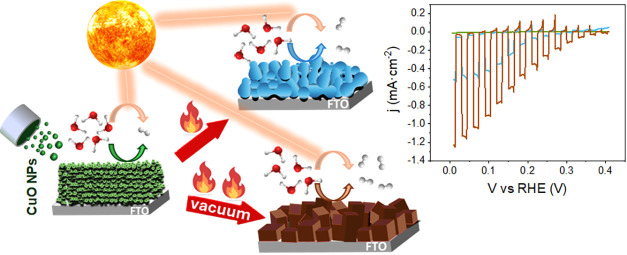

## Introduction

The increasing demand for energy, combined with the environmental impact of fossil fuel emissions and climate change, has driven research and development of sustainable fuel production and clean energy technologies.^1,2^ In this context, photoelectrocatalysis has emerged as a key approach for solar energy conversion, contributing to a cleaner and more efficient energy economy.^3^ Photoelectrodes play a crucial role in facilitating green hydrogen production from water,^4^ CO_2_ photoreduction,^5^ and the degradation of organic pollutants in water.^6^ These processes represent a promising strategy to reduce reliance on fossil fuels and minimize pollutant emissions. To enable these reactions effectively, the development of efficient photoelectrodes is essential.^7^

Photoelectrodes are typically made of semiconductor materials, as their ability to absorb light and drive photoelectrochemical reactions depends on their electronic properties. To achieve efficient solar energy conversion, these materials must have a bandgap that allows absorption within the visible light spectrum. Both photocathodes and photoanodes are essential for completing the redox reactions (reduction and oxidation) required in photoelectrocatalytic processes.^2^ When focusing on semiconductor materials as photocathodes, Cu-based oxides stand out as one of the most employed materials due to their optoelectronic properties, low cost, and abundance.^8^ Among them, copper­(II) oxide (CuO) has emerged as a promising candidate due to its favorable optical and electronic properties. CuO is a p-type semiconductor with a bandgap typically ranging between 1.2 and 1.7 eV, depending on factors such as its crystal structure, defect concentration, and morphology, which are influenced by the fabrication method. These characteristics enable efficient visible-light absorption.^9,10^ The CuO bandgap allows for effective harvesting of solar energy and facilitates redox reactions under ambient conditions. Additionally, its abundance and low cost make it an attractive option for sustainable energy applications.^10^ However, it has some disadvantages: its performance is affected by fast charge carrier recombination, poor photochemical stability under long-term operating conditions, where partial reduction to metallic Cu may occur, and limited charge mobility.^11^ In recent years, nanostructuring strategies have been extensively explored to overcome these limitations by increasing the active surface area, shortening carrier diffusion paths, and promoting efficient charge separation.^12–15^ In addition to these morphological and transport-related advantages, nanoscale CuO can exhibit modified electronic properties, including a slight widening of the bandgap, which enables catalytic processes such as water splitting, a reaction that remains hindered for bulk CuO.^16^ On the other hand, many efforts have been devoted to engineering CuO-based heterostructures, which can significantly improve the photocurrent density and operational stability.^17–23^ Despite the significant progress achieved through these strategies, precise control over morphology, crystallinity, and defect density remains challenging. These parameters critically determine charge carrier transport, surface reaction kinetics, and photostability in photoelectrochemical (PEC) applications. In particular, the fabrication of well-defined CuO nanostructures with optimized charge-transport pathways and stable phase composition remains nontrivial. Therefore, the development of controllable and robust synthetic methodologies capable of delivering highly crystalline, structurally tailored, and reproducible CuO nanoarchitectures is crucial.

To meet these requirements, a wide variety of fabrication routes have been explored for CuO photocathodes.^11^ Wet-chemical methods, including hydrothermal, solvothermal, sol–gel, precipitation, and electrodeposition techniques, offer morphological versatility and relatively low-cost processing.^9,12–15,24,25^ However, achieving precise crystallographic control and minimizing defect density through these approaches can be challenging, often requiring additional postsynthetic treatments. Physical deposition approaches, including sputtering, pulsed laser deposition, chemical vapor deposition, and controlled thermal oxidation, enable the fabrication of highly crystalline CuO films with strong adhesion to conductive substrates.^26,27^ Although these methods provide excellent control over the thickness and stoichiometry, they typically yield relatively compact films or morphologies with limited hierarchical structuring, which may restrict surface-area enhancement and nanoscale defect engineering.

Consequently, despite the extensive toolbox available for CuO synthesis, achieving simultaneous control over the crystal structure, defect density, morphology, and interfacial quality remains challenging in the CuO photocathode fabrication. Specifically, strategies that combine the high structural quality of physically derived CuO with controlled nanoscale structuring are still limited. A highly promising approach is the use of gas-phase synthesis, which offers the advantage of being solvent-free, leaving the whole surface of the nanostructures available for electron transport to the electrolyte. In particular, gas aggregation sources (GAS) are a family of NP fabrication techniques based on the generation of a supersaturated vapor, where nucleation and growth take place.^28^ This vapor can be generated by different means. One of the most popular is based on magnetron sputtering, the so-called sputter gas aggregation source (SGAS). This technique has emerged as an alternative for NP fabrication.^28–30^ It offers several advantages, including precise control over the size and morphology^31,32^ of NPs, high purity in a controlled high or ultrahigh vacuum (UHV) environment, and an environmentally friendly approach due to the absence of solvents.^33,34^

Here, we present an alternative route for the fabrication of CuO-based photocathodes. Nanostructured CuO films were fabricated starting from multilayers of CuO NPs synthesized using SGAS. Despite the crystallinity of the individual CuO NPs fabricated,^34^ there is a need for better conduction through the multilayer. Thus, a series of thermal treatments (TT) on the films at different conditions were performed in order to promote nanoparticle sintering and to tailor the crystalline properties of the whole nanostructured coating,^35^ paving the way for its application in photoelectrocatalysis. The obtained results highlight this strategy for producing CuO nanostructured films as a reliable route to fabricate photoelectrocathodes.

## Experimental Section

### Fabrication of Nanostructured CuO Films

Multilayer copper­(II) oxide nanoparticle films were fabricated using a multiple ion cluster source (MICS) from Oxford Applied Research Ltd.,^36^ a type of SGAS with three 2 in. magnetrons operating in a UHV system (base pressure 1 × 10^–9^ mbar) as shown in Scheme 1 (left panel). In this work, we used one magnetron loaded with a copper target (99.99% purity), argon as the sputtering gas with a total flow rate of 150 sccm, and oxygen, through a dedicated entrance, with a flow rate of 10 sccm, to promote the formation of CuO NPs. We fabricated NPs of two different sizes: large NPs (around 14 nm, see Figure S1a,b), injecting 10 sccm of Ar through the working magnetron and 70 sccm through the others; and small NPs (around 8 nm, see Figure S1c,d), injecting 90 sccm of Ar through the operating magnetron and 30 sccm through the others. In the latter case, the aggregation length was also diminished by 12.5 cm compared to the configuration used for the fabrication of large NPs.^33^ The typical power applied to the magnetron after oxygen injection was 17.5 W. The NP deposition rate was monitored using a quartz crystal microbalance located at the same distance from the MICS exit as the substrates used for NPs collection. The deposited mass over the electrodes was 100 μg/cm^2^ or 50 μg/cm^2^. Depending on the characterization employed, we used different substrates: highly oriented pyrolytic graphite (HOPG) for AFM, fluorine-doped tin oxide (FTO, F:SnO_2_) for XPS and electrochemical measurements, and quartz crystal microbalance (QCM) plate for room temperature water adsorption isotherms.

**Scheme 1:**
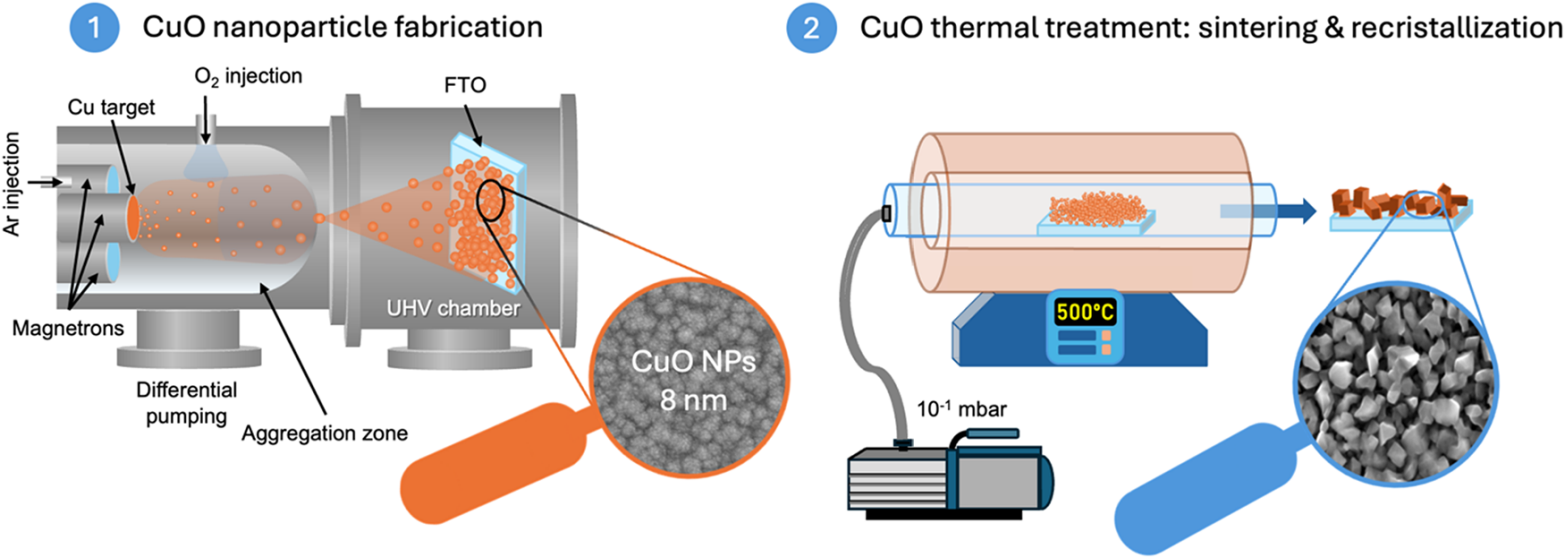
Experimental Setup Used for the Fabrication of CuO Nanostructured Photocathodes, Consisting of Two Stages: (1) Gas-Phase Synthesis of Multilayers of CuO Nanoparticles Followed by (2) Thermal Treatments in a Tubular Furnace to Promote Sintering and Recrystallization

In order to enhance charge transport through the fabricated NP films, we performed a series of thermal treatments on multilayers of CuO NPs onto FTO substrates (see Scheme 1, right panel). The treatments were carried out in a tubular furnace and consisted of a 30 min preheating step at 130 °C, followed by a temperature ramp of 2.5 °C/min up to either 450 or 500 °C. We avoided the use of higher temperatures to prevent damage to the FTO substrates. The target temperature was kept for either 12 or 18 h, after which the temperature was decreased to 130 °C at a rate of 4 °C/min. The thermal treatments were conducted either under an N_2_ atmosphere (TTN) or under vacuum conditions (≈10^–1^ mbar, TTV). The pressure of the TTV was kept high enough to prevent a reduction of the CuO. The samples are named as TTV-X-Y, where ‘TTV’ refers to thermal treatment under vacuum, the middle number indicates the treatment duration (in hours), and the final number corresponds to the temperature (in °C). Table 1 provides a summary of the applied treatments.

**Table 1:** Thermal Treatments Performed on Multilayers of CuO NPs

	thermal treatment (TT)		
	atmosphere	duration (h)	temperature (°C)
TTN	N_2_	12	450
TTV-12–450	Vacuum	12	450
TTV-18–450	Vacuum	18	450
TTV-18–500	Vacuum	18	500

### Characterization Techniques

#### Atomic Force Microscopy (AFM)

AFM measurements were performed by using a Cervantes AFM system from Nanotec Electronica S.L. in dynamic mode. Image analysis was performed using the Gwyddion software.^37^ The size of the nanoparticles was determined by measuring their height when they were deposited on HOPG substrates.

#### Field Emission Scanning Electron Microscopy (FE-SEM)

FE-SEM was performed using a JEOL JSM-7900F microscope equipped with an LED detector, operating at 15 kV and a working distance ranging from 2 to 10 mm. EDX spectra were measured with an ULTIM Max 170 from Oxford Instruments equipped with Aztec software.

#### Transmission Electron Microscopy (TEM)

TEM measurements were performed by using a JEOL JEM 1400 instrument operated at 120 kV. The samples were prepared by mechanically scratching the CuO layer grown on the FTO substrates. The collected powder was subsequently dispersed in isopropyl alcohol (IPA) and sonicated to obtain a homogeneous suspension. Finally, a drop of the resulting dispersion was deposited onto Au TEM grids (Lacey carbon support films, 300 mesh, TED Pella Inc.) and dried at room temperature prior to the measurements.

#### X-ray Diffraction (XRD)

XRD was carried out in a Bruker D8 Advance diffractometer equipped with Cu Kα radiation, a secondary monochromator, and a fast position-sensitive detector (LynxEye).

#### X-ray Photoelectron Spectroscopy (XPS)

XPS of the NPs before and after thermal treatments was performed using a PHOIBOS 100 1D electron/ion analyzer with a 1-DDL detector and a monochromatic Al Kα anode (1486.6 eV). The Cu 2p, Cu_LMM_, and O 1s core levels were recorded with a pass energy of 15 eV and an energy step of 10 meV. The binding energy (BE) was calibrated using the C 1s core level peak at 285 eV from adventitious carbon as a reference. The measurements were carried out ex situ.

#### Photoelectrochemical (PEC) Measurements

PEC characterization was performed in a three-electrode configuration electrochemical cell with a quartz window, using Ag/AgCl (3 M KCl) as the reference electrode, a Pt wire as the counter electrode, and an FTO coated with the nanostructured CuO films as the working electrode (exposed surface: 1 cm^2^).

All of the potentials were referenced to the reversible hydrogen electrode (RHE) using the Nernst equation

$$V_{\mathrm{RHE}}=V_{\mathrm{Ag}/\mathrm{AgCl}}+{V^{0}}_{\mathrm{Ag}/\mathrm{AgCl}}+\left(0.059\;\mathrm{pH}\right)$$

where

$${V^{0}}_{\mathrm{Ag}/\mathrm{AgCl}}\;\left(3\;M\mathrm{KCl}\right)=0.21\;V$$

A PGSTAT204 potentiostat/galvanostat equipped with an integrated FRAII impedance module was used for all PEC measurements. Illumination of the system was performed using a solar simulator (LOT LSH302 Xe lamp equipped with an LSZ389 AM1.5 global filter) calibrated at 1 sun (100 mW·cm^–2^). PEC characterization included linear sweep voltammetry (LSV), chronoamperometry (CA), and electrochemical impedance spectroscopy (EIS. The LSV measurements were performed using chopped illumination with a scan rate of 20 mV·s^–1^, in a 0.1 M potassium phosphate (KPi) buffer solution of pH 7. CA was done at a fixed potential (0.2 V vs RHE) with chopped pulsed light (2 min of darkness and 10 min under illumination). EIS was tested between 0 and 0.4 V each 50 mV using a frequency range between 400 kHz and 100 mHz, in dark conditions and under one sun illumination. For selected LSV and EIS measurements, H_2_O_2_ was added to the electrolyte as an electron scavenger.

The extracted capacitances were calculated from the constant phase element (CPE). The parameters of the CPE, *T*, and *P* are related to the impedance through the equation below, and the ideality factor, *P*, was systematically kept in the range 0.75–1.00.

$$C=\frac{1}{R_{\mathrm{ct}}}{\left(T\cdot R_{\mathrm{ct}}\right)}^{1/P}$$

The charge separation and catalytic efficiencies were calculated using the following equations

$$j_{H2O}=j_{\mathrm{abs}}\cdot \eta_{\mathrm{sep}}\cdot \eta_{\mathrm{cat}}$$

$$j_{\mathrm{HS}}=j_{\mathrm{abs}}\cdot \eta_{\mathrm{sep}}$$

where *j*_H2O_ is the current density in the presence of KPi electrolyte (aqueous), *j*_HS_ is the current density in the presence of H_2_O_2_ as an electron scavenger, *j*_abs_ is the theoretical maximum photocurrent, η_sep_ is the charge-separation efficiency, and η_cat_ is the catalytic efficiency.

The applied bias photon-to-current efficiency (ABPE) has been calculated using the following equation

$$\mathrm{ABPE}\;\left(\%\right)=\frac{\left|j_{\mathrm{ph}}\right|\times \left(1.23-\left|V_{\mathrm{bias}}\right|\right)}{P_{\mathrm{light}}}\times 100$$

where *j*_*ph*_ is the measured photocurrent, *V*_*bias*_ is the applied potential, and *P*_*light*_ is the power of the incident light (1 Sun, 0.1 W/cm^2^).

#### Steady-State and Time-Resolved Photoluminescence (PL and TRPL)

Photoluminescence decay traces were collected by means of the time-correlated single-photon counting (TC-SPC) technique in a Mini-Tau system, using an EPL-375 ps pulsed diode laser with emission at 372 nm as an excitation source (both from Edinburgh Instruments). A band-pass filter centered at 450 nm was employed. The corresponding fitting curves were adjusted by a biexponential function, calculating the average lifetime using the following equation

⟨τ⟩={∑(Aiτi)2}/∑(Aiτi)

where τ is the average fluorescence lifetime, and τ*i* and *Ai* correspond to the lifetime and the pre-exponential value for each species.

#### Transient Absorption Spectroscopy (TAS)

TAS measurements were carried out using an LP980 laser flash equipment from Edinburgh Instruments based on an optical parametric oscillator pumped by the third harmonic of a Nd:YAG laser (EKSPLA). 355 nm was employed as the excitation wavelength with single low-energy pulses of 500 μJ per pulse. For the transient absorption experiments, a pulsed xenon flash lamp (150 W) of 5 ns duration was employed. The probe light was dispersed through a monochromator (TMS302-A, grating 150 lines·mm^–1^) after passing through the sample and was then directed to a PMT detector (Hamamatsu Photonics) to obtain the temporal profile. All transient spectra and kinetics were recorded at room temperature using 1 × 1 cm quartz cells, introducing a thin-film sample into the cuvette. Aqueous potassium phosphate 0.1 M (pH 7) was used as solvent, which was bubbled for 15 min with N_2_ before acquisition.

#### Room Temperature Water Adsorption Isotherms

Adsorption isotherms were obtained by the Quartz Crystal Microbalance method. During the experiment, the temperature of the microbalance was kept constant at 19.0–19.5 °C. Before the acquisition, the QCM-deposited crystal was gently warmed under vacuum (10^–6^ mbar) above 95 °C, then exposed to saturated water vapor, repeating the process to avoid chemisorption and to remove condensed water from the pores. The total pretreatment time lasted 24 h. It must be noted that such characterization is herein only intended for qualitative comparison between as-deposited and treated NPs, and the isotherms are presented normalized at the maximum thickness acquired by the QCM under saturation conditions.^38,39^

## Results and Discussion

As a first step to produce nanostructured CuO films, we carried out an optimization of the fabrication parameters in order to generate CuO nanoparticles (NPs) with average sizes of 8 and 14 nm, hereafter referred to as small and large NPs, respectively (see Figure S1 for the analysis of the NP size). Aiming to fabricate CuO-based photocathodes, multilayers of these two types of CuO NPs were deposited on top of FTO substrates until a total thickness of around 2.3 ± 0.1 μm was achieved (Figure 1a,b). On the other hand, Figure 1c,d display representative top-view FE-SEM images of these multilayer structures. An agglomeration of the NPs was observed, which manifested as noticeable surface protrusions across the samples (Figures S2a–c and S3a–c). This morphology became increasingly evident with smaller NPs.

**Figure 1:**
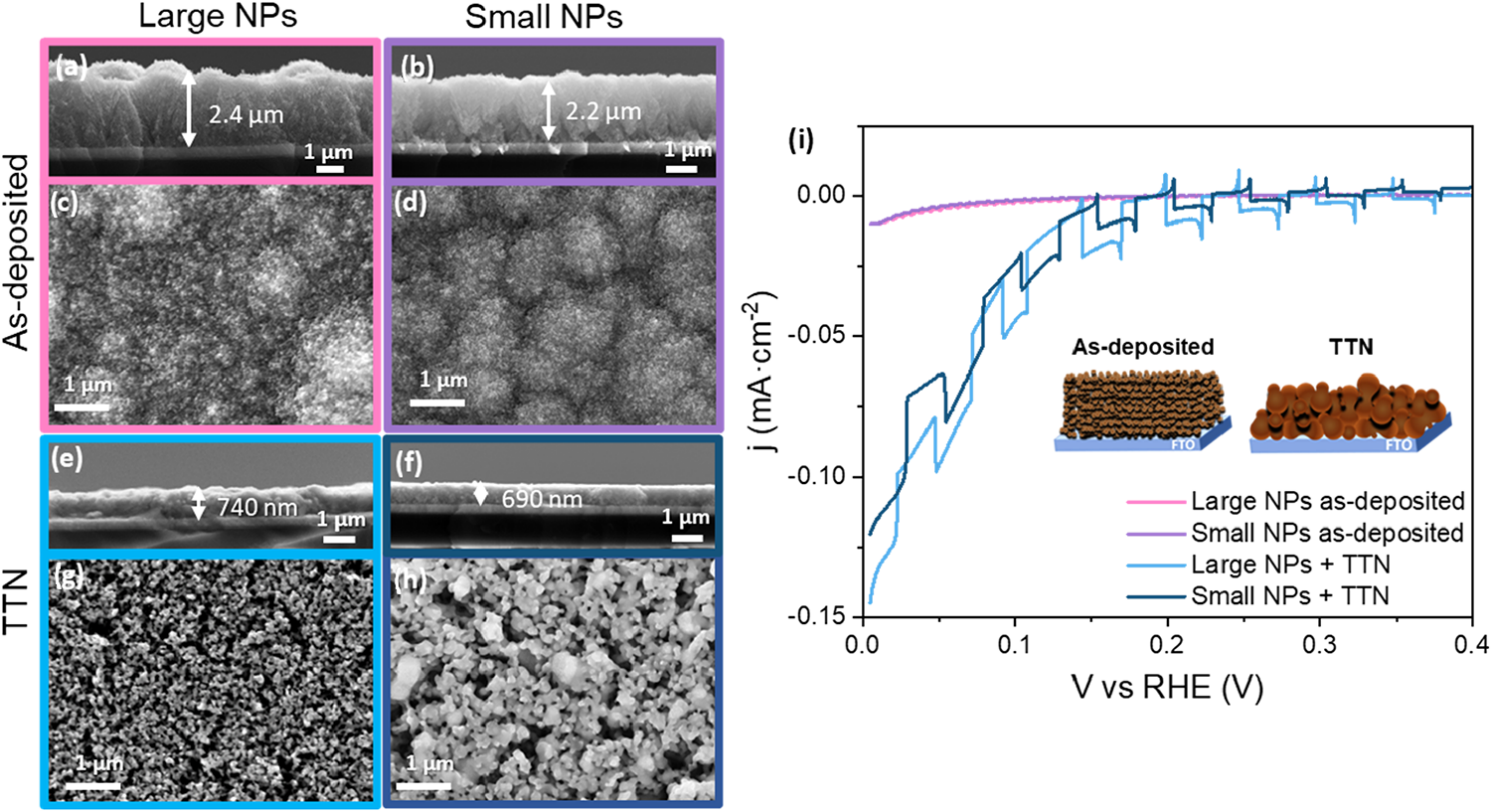
FE-SEM images in cross-section and top view. As-deposited (a, c) large and (b, d) small CuO NPs and after TTN for 12 h at 450 °C (e, g) large and (f, h) small CuO NPs on FTO substrates. (i) LSV curves of the electrodes under 1 Sun chopped illumination in 0.1 M KPi electrolyte. Inset showing a scheme of the CuO NPs as-deposited and after TTN.

The photoelectrochemical response of the as-deposited CuO photocathodes was tested by means of linear sweep voltammetry (LSV) (Figure 1i, pink and violet traces), with negligible registered PEC activity. This effect could be related to the difficulty of achieving effective charge conduction across the CuO NP multilayer. It must be taken into account that, even though these CuO NPs are crystalline,^34,40^ they are randomly oriented. The electrical conduction through this kind of nanogranular film is quite complex even in the case of metallic clusters due to the large number of interfaces, grain boundary junctions, and crystal lattice defects,^41,42^ which usually act as recombination centers.^43^

Thus, we performed different thermal treatments to enhance conduction through the films and improve their PEC performance, with special care to avoid damaging the FTO substrate. The first thermal treatment carried out in a nitrogen atmosphere for 12 h at 450 °C (TTN) induced a clear modification of the morphology of the films with a final thickness of one-third of the original value (see cross sections in Figure 1e,f) and bigger domains clearly visible in Figure 1g,h. This effect could be related to an Ostwald ripening process (OR) through the transfer of atoms or molecules from smaller to larger particles to minimize the surface energy of the NPs.^44^ As a result, the TTN promoted a partial sintering of the NPs (Figures S2d–f and S3d–f) and gave rise to the formation of more porous nanostructured films.

Nevertheless, there were some observable differences among the films. Larger domains were obtained after TTN when starting from smaller as-deposited NPs (Figure 1h), as the OR process increased with the decreasing NP size.^45^ Temperature also has a role in this process due to its effect on the interfacial energy.^46^ In this case, even though the temperature of the TTN was the same for both systems, it is well-known that the melting point is size-dependent.^47–49^ Consequently, larger NPs presented a less pronounced coalescence (Figure 1g), and they are less likely to fuse together.^50^

The PEC response of the thermally treated electrodes (light and dark blue traces in Figure 1i) evidenced that the modifications induced on the CuO NP films with TTN produced a positive effect in the generated photocurrent. However, the collected currents were in the order of −0.03 mA·cm^–2^, and no significant differences between the two types of nanostructured coatings tested were observed.

In order to maximize the generated photocurrent, we optimized the fabricated electrodes by adjusting the size of the NPs, their load, and the thermal treatment. With this purpose, we selected the small NPs, as they promoted a more efficient formation of larger domains in the film. In addition, we reduced the load of CuO NPs by half (from 100 to 50 μg/cm^2^), as the higher load did not lead to an improvement in the performance of the electrodes (unpublished results). Finally, seeking a more effective thermal treatment, we modified (see Table 1): (i) the atmosphere, from nitrogen to vacuum (TTV), although nitrogen is relatively inert, vacuum conditions are expected to further reduce contamination and enhance atomic mobility; (ii) the temperature, from 450 to 500 °C, a careful selection to balance improved coalescence while preserving the integrity of the FTO electrode; and (iii) the treatment duration, from 12 to 18 h, to leave enough time for growth processes to occur. As we already mentioned, the samples are named as TTV-X-Y, with X being the treatment duration (in hours) and Y the temperature (in °C).

Figure 2 displays the resulting CuO morphology after TTV-12–450, TTV-18–450, and TTV-18–500 treatments. Just by changing the TT atmosphere to vacuum (TTV-12–450, Figure 2a), we observed a denser coating with the absence of surface channels compared to TTN (Figure S4c vs Figure S3e), as expected from a more efficient process due to the absence of conduction and convection heat transfer. Extending the TTV duration from 12 to 18 h (TTV-18–450, Figure 2b) led to the onset of incipient crystallization, with some observable geometric features (see inset). At this stage, the growth mechanism changed to a coalescence of neighboring NPs, with the occurrence of bridges between them and the formation of non spherical assemblies.^51^ Therefore, with longer treatment times, the system had enough time to evolve, as this TTV-18–450 film has a more compact appearance than TTV-12–450. This fact was confirmed by the cross-section (Figure S4a vs S4e), where the thickness was slightly above a quarter of the as-deposited thickness. Finally, by increasing the treatment temperature (TTV-18–500, Figure 2c), the recrystallization of the NP film was complete with the formation of cubic-shaped nanodomains of larger size (edges around 200 nm) and no traces of the original NPs. The resulting film was thinner than the ones fabricated with the other TTVs (see cross sections in Figure S4a,e,i). The larger porosity of TTV-18–500 as compared to that of as-deposited NPs is further supported by the adsorption isotherms carried out by the QCM method (see Figure S5). Both isotherms exhibit the characteristic Type IV profile, confirming that mesopores (2–50 nm) dominate the pore structure. The as-deposited NPs, however, show a higher relative fraction of micropores (pore width below 2 nm), as indicated by the larger adsorbed volume at low partial pressures. Moreover, the desorption branch of the isotherm for the as-deposited NPs displays a slight hysteresis opening, consistent with the presence of ink-bottle-type pores and/or minor swelling or contraction of pore apertures during the adsorption–desorption cycle, likely related to a lower degree of sintering for untreated nanoparticles. It should also be noted that pore sizes above this range, particularly macropores with widths exceeding 50 nm, cannot be uniquely resolved by this technique. Even so, the differences observed in the desorption branches indicate a more open porosity in the TTV-18–500 system, which facilitates more complete pore evacuation.^39^

**Figure 2:**
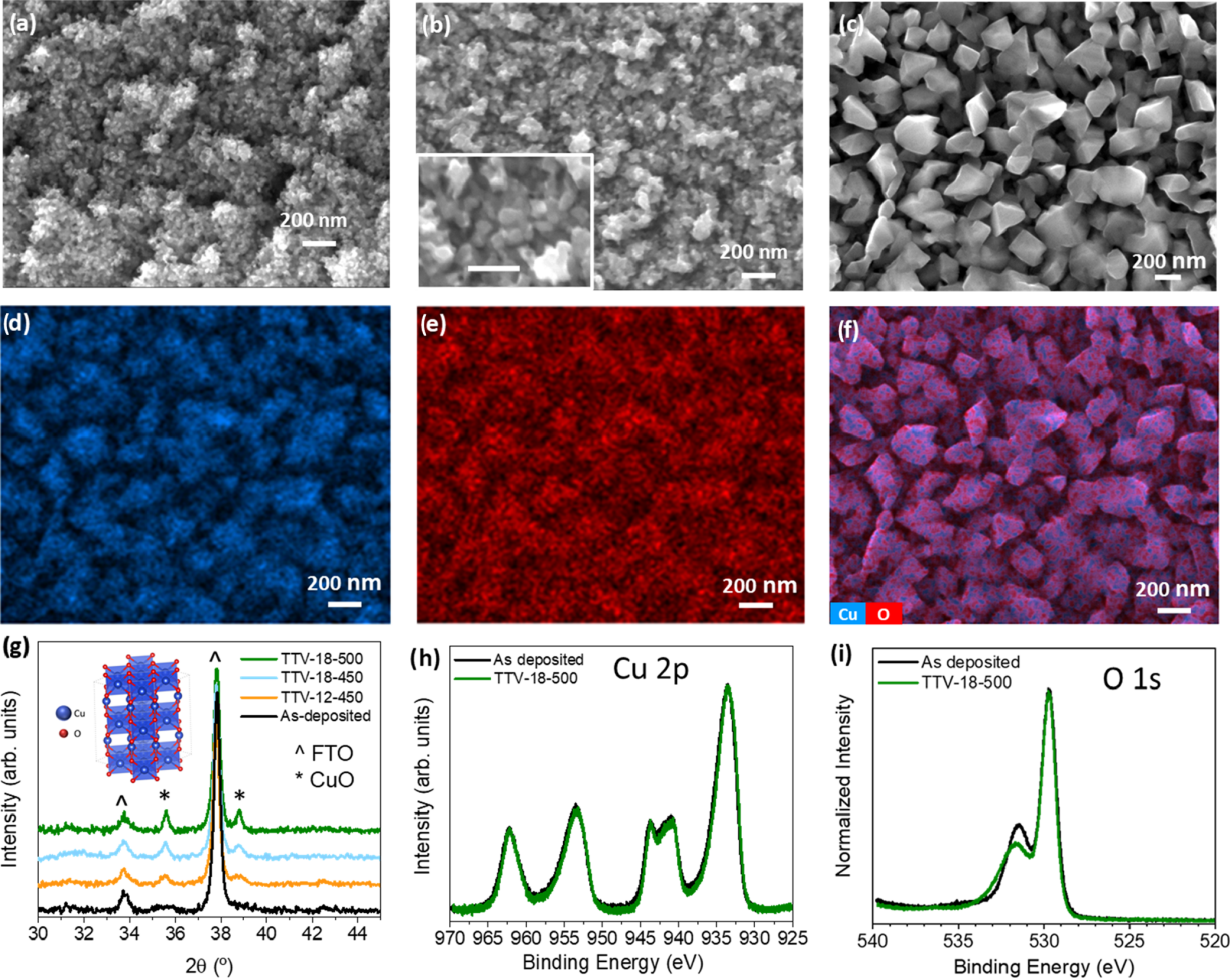
FE-SEM images of the different CuO electrodes after (a) TTV-12–450, (b) TTV-18–450, and (c) TTV-18–500. EDX mapping of the image depicted in (c): (d) copper Lα1,2, (e) oxygen Kα1, and (f) overlap of mappings (d) and (e). (g) XRD patterns of as-deposited NPs and after different TTVs. Inset shows the simulated monoclinic CuO structure with Cu atoms in blue and oxygen atoms in red. (h) Cu 2p and (i) O 1s XPS core level spectra of as-deposited NPs and after TTV-18–500. The scale bar of the inset in (b) is 200 nm.

EDX mapping displayed in Figure 2d–f revealed the homogeneous presence of copper and oxygen at the surface. Figure S6 presents an EDX spectrum of sample TTV-18–500 with signals arising from copper and oxygen from the nanostructured coating, tin of the FTO substrate, silicon from the glass, and a small fraction of carbon from atmospheric contamination.

In order to have a deeper insight into the crystalline changes of the nanostructured CuO layers induced by TTVs, we carried out an XRD analysis of these samples (Figure 2g). The two most intense diffraction peaks at 37.8° and 33.8° (marked with a pyramid) emerged from the FTO substrate (see also Figure S7), while the emissions at 35.6° and 38.8° (black asterisks) corresponded to the CuO film. For the as-deposited electrode (black curve), only FTO peaks were evident in the diffractogram, while the small feature around 35.5° likely arises from the CuO layer. After TTV-12–450 and TTV-18–450 (orange and blue curves), where different degrees of coalescence and recrystallization were taking place within the layer, monoclinic CuO (see inset structure in Figure 2g) diffraction peaks started to appear at 35.6° and 38.8°, corresponding to (111) and (002) planes, respectively.^52^ Once the CuO nanocubes were formed (TTV-18–500, green curve), these two diffraction peaks were neat, confirming the formation of a crystalline CuO structure along the electrode after TTV-18–500. Using the Debye–Scherrer equation (see Table S1), we evaluated the mean crystallite size. There is an increase of these CuO crystallites by a factor of 1.3 when extending the TTV from 12 to 18 h and a further 2-fold increase when increasing the temperature from 450 to 500 °C, reaching mean sizes of 45 nm with TTV-18–500. Therefore, the XRD results confirm the coalescence of CuO NPs to form larger CuO nanostructures (TTV-12–450 & TTV-18–450) and the formation of bigger CuO nanocubes after TTV-18–500.

Transmission electron microscopy (TEM) was further employed to gain deeper insight into the microstructural evolution of the CuO nanostructured films upon thermal treatment (Figure S8) on the two samples with the most different TT conditions (TTV-12–450 and TTV-18–500). The TEM analysis revealed a clear increase in nanocrystal size when comparing both treatments, consistent with a progressive coalescence and recrystallization process induced by the more intense thermal treatment. This evolution leads to the formation of larger and better-defined CuO nanodomains, in excellent agreement with the increase in crystallite size and improved crystallinity observed by the XRD analysis.

Possible surface reduction of the outermost surface after TTV-18–500 was evaluated by measuring the Cu 2p XPS core level spectra before and after the thermal treatment (Figure 2h). There is an overlap of both spectra with the main emission corresponding to the Cu 2p_3/2_ signal at around 933.5 eV and a shakeup satellite at around 940.9 eV, both characteristic of Cu^2+^.^34,53^ The shape of the satellite peak together with the Cu_LMM_ Auger spectra (Figure S9), evidence that copper is in the form of CuO,^34,53^ with no clear changes after the treatment that would infer a significant contribution of other compounds (i.e., Cu­(OH)_2_ or reduction to Cu_2_O). In contrast, the O 1s XPS spectra (Figure 2i) exhibit two main contributions. The dominant component, centered at 529.7 ± 0.1 eV, is assigned to lattice oxygen (O^2–^) in the CuO structure. A second contribution appears at higher binding energies, where signals may originate from different oxygen environments. In our case, this component lies at 531.5 ± 0.1 eV and is commonly attributed to nonlattice oxygen species, including oxygen atoms in defective or under-coordinated environments and surface-related oxygen species such as hydroxyl groups or weakly adsorbed oxygen-containing species, among others.^54–56^ It should be noted that the amount of oxygen-containing species from adventitious carbon represents only around 5% of the sample (extracted from the analysis of the C 1s core level peak, not shown here). The high-BE component, which is characteristic of nanostructured materials with a high surface-to-volume ratio, undergoes a noticeable broadening after the thermal treatment. The broadening of this component is attributed to an increased distribution of nonequivalent oxygen environments at the surface, arising from the generation of oxygen-related defects and oxygen vacancies during vacuum annealing, as well as to the stabilization of surface hydroxyl species at these defective sites upon exposure to ambient conditions. One has to keep in mind that, as the measurements were performed ex situ, any surface oxygen vacancy created during vacuum annealing would be healed upon exposure to ambient conditions.^55–58^ Importantly, this evolution of the O 1s signal occurs without detectable changes in the copper chemical state, indicating that the thermal treatment primarily affects the oxygen sublattice rather than inducing copper reduction or hydroxide formation.

Thus, the photoelectrochemical performance of the fabricated photocathodes was analyzed. Figure 3a shows the j–V response of these new nanostructured CuO films in a 0.1 M KPi buffer (pH = 7) electrolyte, with photogenerated currents up to 1 order of magnitude higher than those obtained with the TTN electrodes (Figure 1i), along the entire potential window. As expected, increasing CuO crystallinity under progressively more intensive TTV conditions (TTV-12–450 < TTV-18–450 < TTV-18–500) resulted in enhanced photocurrents, obtaining a maximum photocurrent density of −1.2 mA·cm^–2^ after TTV-18–500, followed by TTV-18–450 with −0.75 mA·cm^–2^ and TTV-12–450 with −0.5 mA·cm^–2^. These values are in the range of other bare nanostructured CuO photoelectrodes reported in the literature, like CuO branched nanowires with a photocurrent density of −1 mA/cm^2^ at 0 V vs RHE (electrolyte 0.25 M Na_2_SO_4_, pH = 5.70)^14^; electrodeposited CuO with generated current densities between −0.39 and −0.43 mA cm^–2^ at −0.55 V vs Ag/AgCl (1 M KOH), depending on the film thickness^23^; hydrothermal synthesized CuO with photogenerated current densities of −0.7 mA cm^–2^ at 0 V vs RHE (0.1 M Na_2_SO_4_, pH = 6.89)^22^; spin-coated CuO nanoparticles that generated between – 1.2 and – 1.58 mA/cm^2^ at −0.55 V vs Ag/AgCl (1 M KOH, pH = 14);^12,22^ or CuO nanoleaves with photocurrents of −1.1 to −1.5 mA/cm^2^ at 0 V vs RHE (0.1 M Na_2_SO_4_, pH = 5.8).^15^

**Figure 3:**
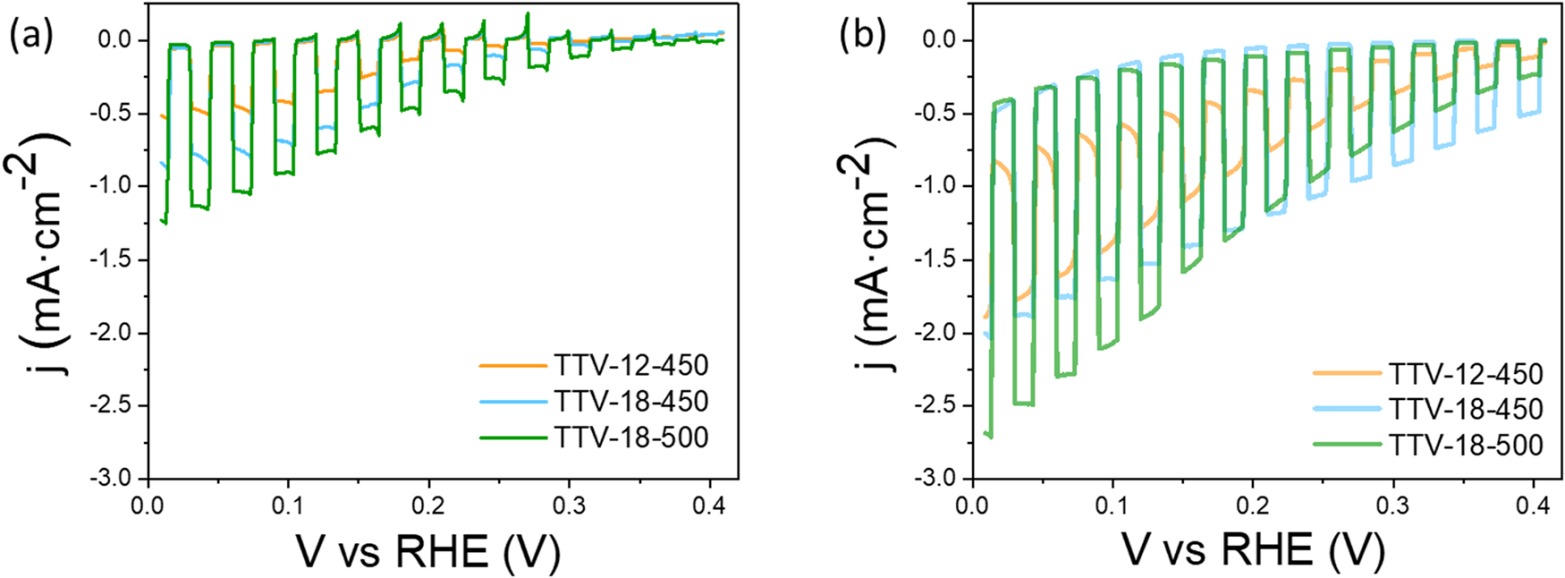
LSV curves under 1 Sun chopped illumination of the samples after TTVs (a) in 0.1 M KPi electrolyte and (b) in 0.1 M KPi electrolyte and H_2_O_2_ as an electron scavenger.

Trying to obtain the maximum performance of the fabricated photocathodes, their PEC response was also evaluated in the presence of an electron scavenger. Specifically, a similar trend in the LSV measurements was observed when adding H_2_O_2_ to the KPi electrolyte (Figure 3b). The higher photocurrents registered in comparison to Figure 3b were related to the presence of H_2_O_2_, acting as an electron scavenger (note the absence of recombination) and leading to photocurrents up to −2.7 mA· cm^–2^ for the TTV-18–500 electrode. From this point onward, we will focus on the samples fabricated under the most distinct thermal treatment conditions, namely, TTV-12–450 and TTV-18–500. Additionally, to further evaluate the stability of the fabricated photocathodes, chronoamperometric measurements were carried out under continuous illumination (Figure S10), comparing the optimized TTV-18–500 electrode with the less-treated TTV-12–450 sample. As expected from the j–V curves, the TTV-18–500 photocathode exhibits a significantly higher initial photocurrent for hydrogen evolution. However, both electrodes display a similar stability trend over time. In particular, the photocurrent progressively decreases during operation, reaching approximately one-quarter of the initial value after 4000 s and stabilizing around −0.1 mA·cm^–2^. This behavior is consistent with the known photocorrosion issues of CuO-based photocathodes in aqueous electrolytes, yet can still be considered a reasonably stable performance within this material family. The comparable stability observed for both samples suggests that the degradation mechanism is not strongly dependent on the specific morphology but rather on surface processes. We attribute this behavior to the relatively compact nature of the thermally treated films, which likely limits electrolyte penetration into the bulk of the electrode. As a result, photocorrosion is expected to occur predominantly at the surface, leading to partial reduction of CuO to Cu_2_O. This interpretation is supported by post-PEC characterization (XRD and XPS), shown in Figures S11 and S12, which indicates a partial reduction to Cu_2_O. Xing et al.^23^ already reported a similar evolution of XRD and XPS measurements and concluded that the CuO photocathode photocorrosion was mainly caused by this self-reduction of CuO into Cu_2_O. These results highlight that, while morphology strongly impacts photocurrent generation, the overall stability remains governed by surface chemical transformations under operating conditions.

Other complementary experiments were performed to further understand the enhanced PEC performance of the higher-temperature treatment. As shown in Figure 4a, the TTV-18–500 sample exhibited higher optical absorbance values than the TTV-12–450 photocathode across the entire investigated wavelength range, indicating a more efficient light harvesting capability after the more intensive thermal treatment. In order to further evaluate the optical properties of the CuO films, Tauc plots (Figure S13), assuming an indirect optical transition, were calculated from the absorbance spectra. The extracted optical bandgap energies were approximately 1.35 eV for the TTV-18–500 photocathode and 1.39 eV for the TTV-12–450 sample, both in very good agreement with previously reported values for CuO in the literature.^59^ The slightly narrower bandgap obtained for the TTV-18–500 sample is consistent with its enhanced optical absorption and contributes to its improved PEC performance, as a broader portion of the solar spectrum can be effectively absorbed and converted into photogenerated charge carriers.

**Figure 4:**
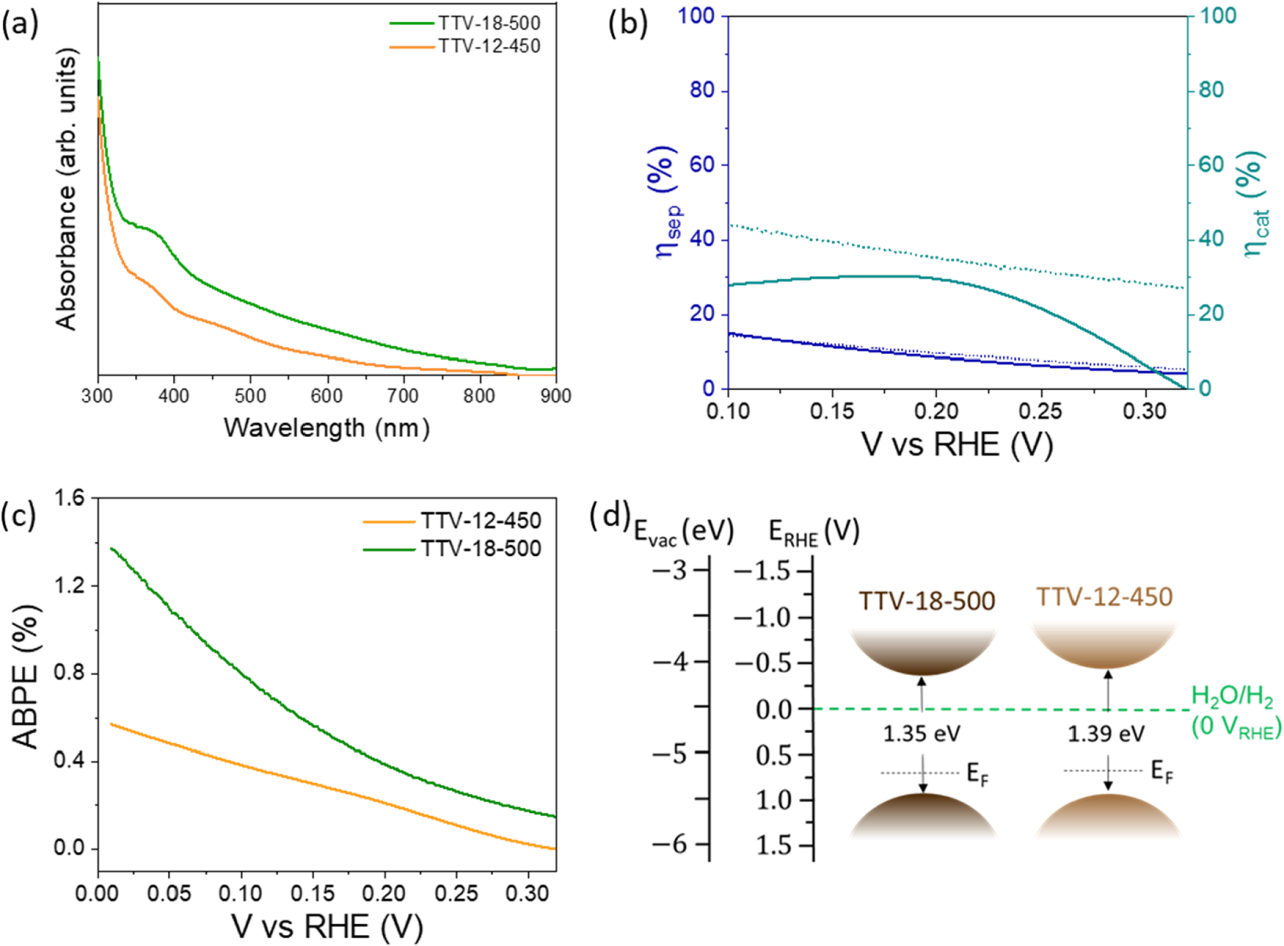
(a) Optical absorbance of the analyzed samples. (b) Charge separation (η_sep_) and catalytic (η_cat_) efficiencies calculated for the TTV-12–450 (dotted lines) and TTV-18–500 (continuous lines). (c) Calculated ABPE (%) for both analyzed samples under 1 Sun illumination. (d) Band energy diagram of the fabricated photocathodes.

On the other hand, Figure 4b shows the charge-separation efficiency (η_sep_) and catalytic efficiency (η_cat_) of the fabricated CuO photocathodes as a function of the applied potential. The η_sep_ values ranged between approximately 10 and 20% for both samples, with slightly higher values obtained for the TTV-18–500 photocathode along the whole potential window. This behavior indicates that the more extensive recrystallization and coalescence induced by the thermal treatment at higher temperature contribute to a moderate improvement in charge separation and reduced bulk recombination losses. More significant differences were observed for the catalytic efficiency. While the η_cat_ of the TTV-12–450 sample tends to saturate slightly below 30%, the TTV-18–500 photocathode exceeded 40% and remained consistently higher throughout the entire investigated potential range. These results suggest that the formation of larger and more crystalline CuO nanodomains after the 500 °C treatment promotes a more efficient interfacial charge transfer and catalytic reduction reaction at the CuO/electrolyte interface. Overall, the simultaneous enhancement of both η_sep_ and η_cat_ is in good agreement with the improved PEC response observed in the j–V measurements.

To further evaluate the practical PEC performance of the fabricated photocathodes, the applied bias photon-to-current efficiency (ABPE) was calculated (Figure 4c). The ABPE parameter reflects the overall efficiency of converting solar energy to chemical energy under an externally applied bias. In both samples, the ABPE increased with increasing cathodic bias, reaching maximum values at 0 V vs RHE. The TTV-12–450 photocathode achieved a maximum ABPE of approximately 0.6%, whereas the TTV-18–500 sample reached significantly higher values of up to 1.4%. The maximum ABPE values obtained in this work, reaching up to 1.4% for the TTV-18–500 photocathode, are higher than those of many previously reported bare CuO-based photocathodes, even with more complex architectures, operating in aqueous electrolytes.^21,60,61^ These results further confirm the beneficial impact of the optimized thermal treatment and the enhanced crystallinity of the CuO nanostructures on their overall PEC performance.

The band energy diagram of the analyzed CuO-based photocathodes (Figure 4d) was constructed by combining experimental measurements with literature-derived values. Specifically, the band alignment was determined using XPS measurements (providing the energy difference between the valence band maximum and the Fermi level shown in Figure S14), Tauc plot analysis (for the optical bandgap), and valence band positions reported in the literature.^8,62^ As it can be observed, TTV-12–450 exhibits a slightly higher conduction band minimum compared to TTV-18–500, which may in principle provide a modest increase in the thermodynamic driving force for the hydrogen evolution reaction by enhancing the reducing power of photogenerated electrons. However, this difference is marginal and does not translate to improved PEC performance. Instead, the overall photoelectrochemical response is governed by other dominant factors, where TTV-18–500 clearly outperforms TTV-12–450, including its narrower bandgap, higher light absorption, and improved catalytic activity and charge-separation efficiency. Collectively, these advantages lead to significantly enhanced PEC performance, despite the slightly less favorable conduction band alignment.

To further understand the mechanisms behind the PEC performance of the fabricated CuO TTV-12–450 and TTV-18–500 photocathodes, EIS measurements were performed. Figure 5a exhibits the Nyquist plots under dark and 1 Sun conditions at 0.0 V vs RHE. As can be observed, a single arc was observed on both samples, suggesting the presence of a single time constant. Therefore, a simple Randles’ circuit with a constant phase element (CPE) (inset Figure 5b) was employed to fit the raw data.^63^ The series resistance (*R*_s_) values account for the resistance of the entire setup, including the electrolyte, wiring, potentiostat, and FTO substrate. The *R*_s_ values of the fitting remained close to 55 Ω·cm^2^ throughout the entire tested potential window (Figure 5b), in good agreement with previous studies using the same experimental setup.^64^

**Figure 5:**
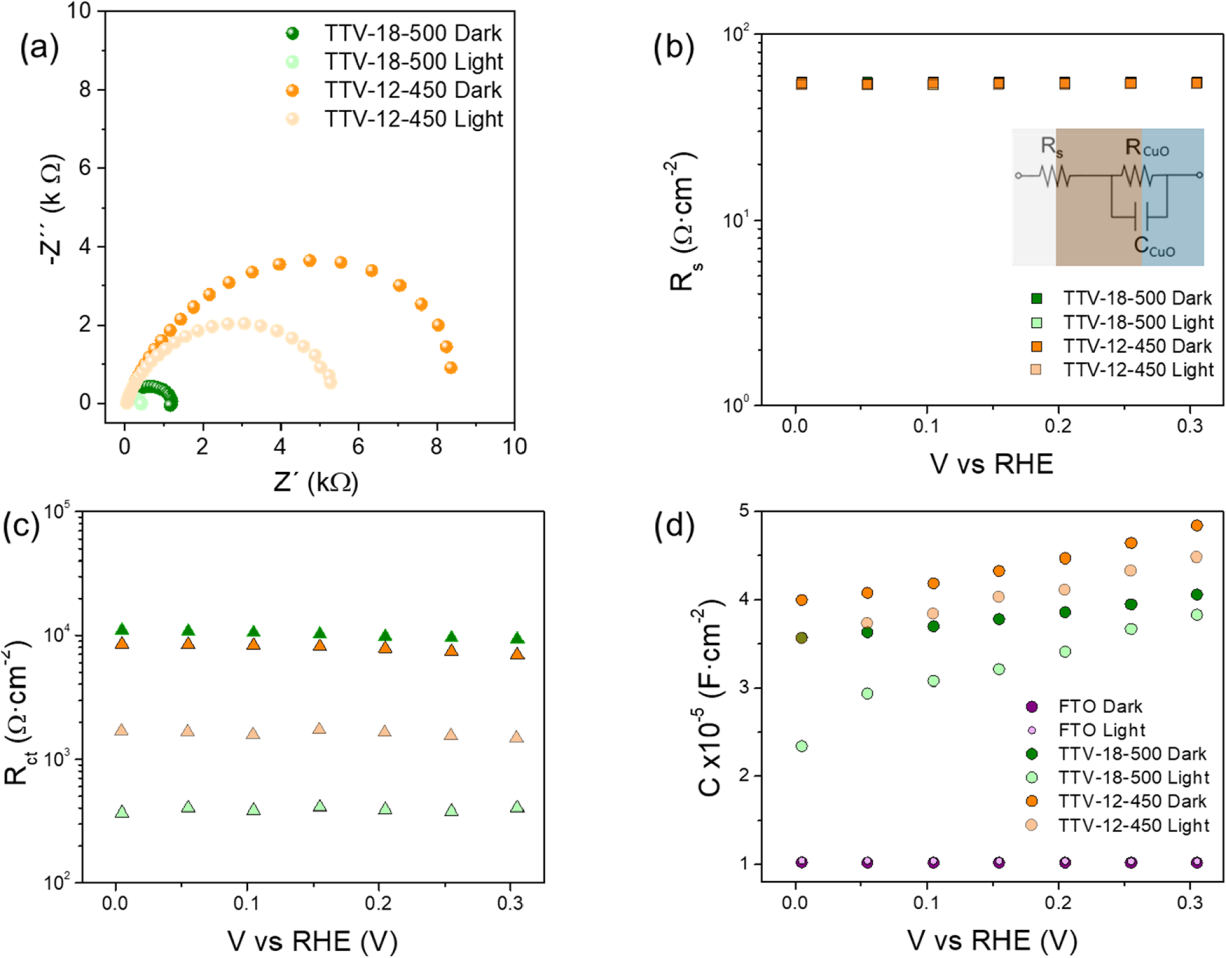
EIS of TTV-12–450 (orange) and TTV-18–500 (green) electrodes for light (hollow) and dark (full) conditions for a potential window from 0.4 to 0.0 V (vs RHE) every 50 mV in 0.1 M KPi electrolyte + H_2_O_2_. (a) Nyquist plot at 0.0 V vs RHE, (b) series resistance (*R*_s_), (c) charge-transfer resistance (*R*_ct_), and (d) capacitance (C) values extracted from the employed Randles’ equivalent circuit, with a CPE, used for the fitting of the raw data (see inset in b).

On the other hand, the parallel R-C couple represents the electrical properties of the CuO electrode–electrolyte interface. *R*_ct_ represents the charge-transfer resistance between the CuO photocathode and the electrolyte (Figure 5c). *R*_ct_ was found to decrease under illumination in both samples, as expected in a semiconductor.^65,66^ It is important to notice that the TTV-18–500 sample shows lower values of *R*_ct_ both under dark and under illumination conditions, as well as a higher reduction of the resistance upon illumination, than the TTV-12–450 sample, in good agreement with the PEC response shown in Figure 3. Finally, the capacitance (C) element of the equivalent circuit (Figure 5d), extracted from the CPE, represents the charge accumulation at the interface between the nanostructured CuO film and the electrolyte. The larger capacitance values of the TTV-12–450 sample along the whole potential window can be attributed to a more difficult extraction of the photogenerated electrons, which accumulate at the interface. In contrast, the lower capacitance values of the TTV-18–500 electrode indicated a more effective charge transfer. In addition, the extracted capacitances were found to decrease, both under dark and under illumination, with the applied cathodic bias, as an indication of better charge extraction of the photogenerated electrons to the electrolyte.^67^ Note that this decrease in the capacitance with the light and with the cathodic potential is higher in the TTV-18–500 photocathode, which is in good agreement with the higher PEC performance of this sample. Also, the extracted capacitances of the bare FTO substrate are shown in Figure 5d for comparison. FTO is a degenerate semiconductor, behaving as a metal; therefore, its capacitance remains constant with the applied potential and under illumination.

Going one step further in the understanding of the charge dynamics, photoluminescence (PL) (Figure 6a), time-resolved photoluminescence (TRPL) (Figure 6b and Figure S15), and transient absorption spectroscopy (TAS) (Figure 6c,d) experiments were performed on the analyzed photocathodes. Figure 6a shows the PL spectra of the TTV-12–450 sample (orange traces) resulted in a broad band from 450 to 550 nm exhibiting a photoluminescence lifetime (τ_PL_) of 0.85 ns.^68,69^ Comparatively, the radiative emission of the TTV-18–500 sample (green traces) displayed lower intensity and increased lifetime to 1.28 ns,^70^ which indicated a better charge separation, and a reduced charge recombination, for the TTV-18–500 sample.

**Figure 6:**
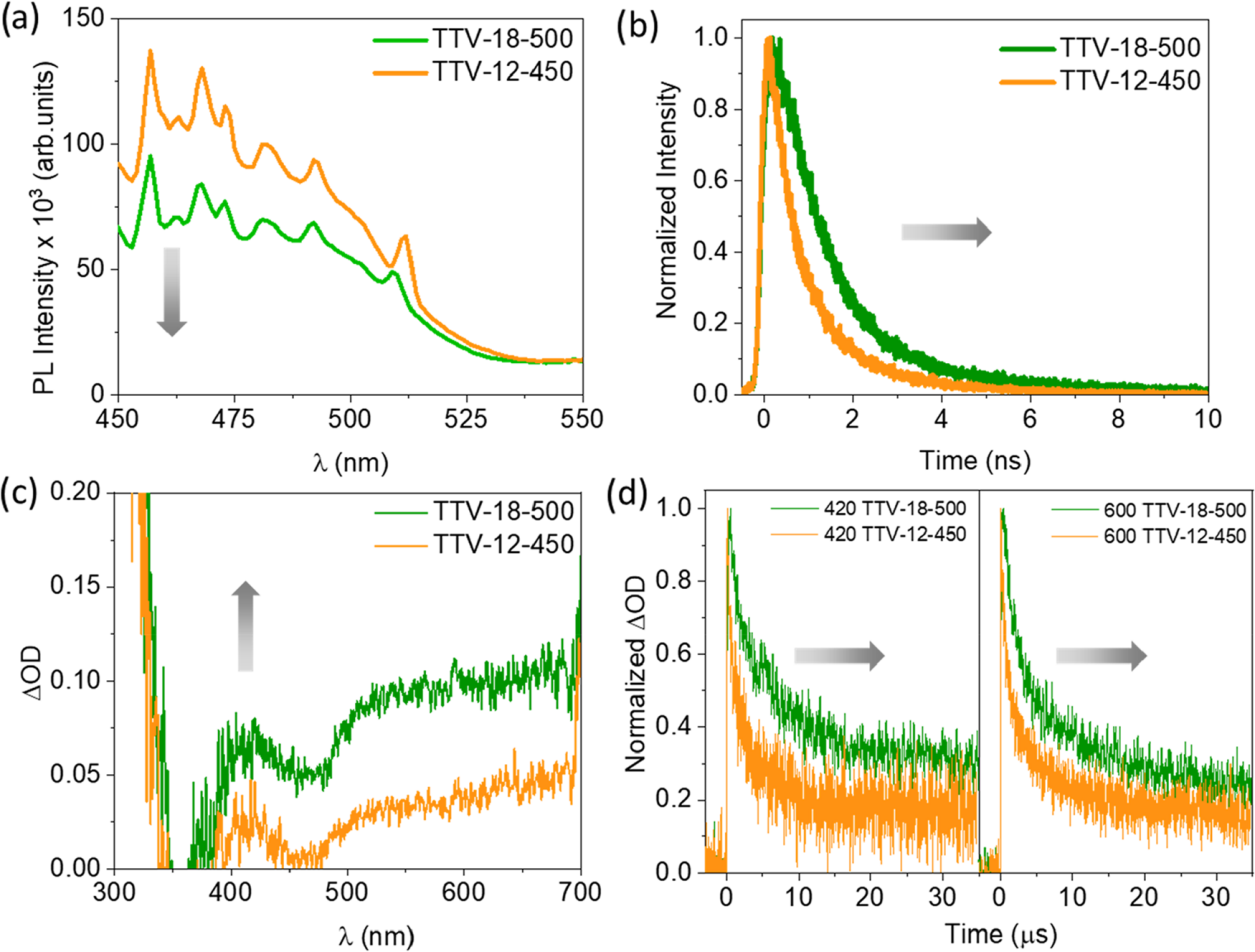
(a) Photoluminescence spectra (λ_exc_ = 350 nm) for the TTV-12–450 (orange) or TTV-18–500 (green). (b) Photoluminescence decay traces (λ_exc_ = 372 nm, band-pass filter between 475 and 525 nm) for the TTV-12–450 (orange) or TTV-18–500 (green). (c) Transient absorption spectra (λ_exc_ = 355 nm) for the TTV-12–450 (orange) or TTV-18–500 (green). (d) Transient absorption decay traces (λ_exc_ = 355 nm, λ_obs_ = 420 or 600 nm) for the TTV-12–450 (orange) or TTV-18–500 (green).

The TAS spectra of deaerated films (Figure 6c) exhibited two absorption features: a band centered at 400–450 nm and another extending beyond 500 nm, associated with the photogeneration of holes and electrons, respectively.^71,72^ Interestingly, the TAS of the TTV-18–500 sample (green trace) increased in all spectral windows (from 400 to 700 nm), indicating a higher population of long-lived charge carriers and thus more efficient charge separation. This improvement is also reflected in the transient lifetimes (τ_TAS_): while the TTV-12–450 sample exhibited lifetimes in the range of 2.7–3.2 μs, the τ_TAS_ of the TTV-18–500 sample nearly doubled, reaching 6.5–8 μs (Figure 6d and Figure S16). Thus, a 2.5-fold increase was observed in the photogenerated electrons’ lifetime at the water reduction time scale in correlation with the enhanced PEC performance of the TTV-18–500 sample.

Taken together, the PL, TRPL, and TAS analyses consistently demonstrate that the TTV-18–500 electrode undergoes a substantial reduction in electron–hole recombination, resulting in significantly improved carrier dynamics, in good agreement with the EIS results showing a reduced charge-transfer resistance and capacitances observed in the TTV-18–500 photocathode.

In summary, we explored a new route for the synthesis of CuO photocathodes. Nanostructured CuO films were fabricated through a two-step process. First, multilayers of CuO NPs were synthesized via a sputter gas aggregation source. In a second stage, the multilayers underwent thermal treatments under varying temperatures, durations, and conditions, modifying the film morphology to reach enhanced photocatalytic activity. Scheme 2 presents a schematic of this process. The observed evolution of the morphology of the fabricated nanostructured films suggested that, starting from the as-deposited CuO NPs (stage 1), there was an initial growth of the NPs (Ostwald ripening) followed by a partial coalescence with the surrounding NPs (stage 2) with thermal treatments. This process preceded crystallization and the formation of CuO nanocubes (stage 3), provided that the system had enough time and temperature for these phenomena to occur.

**Scheme 2:**
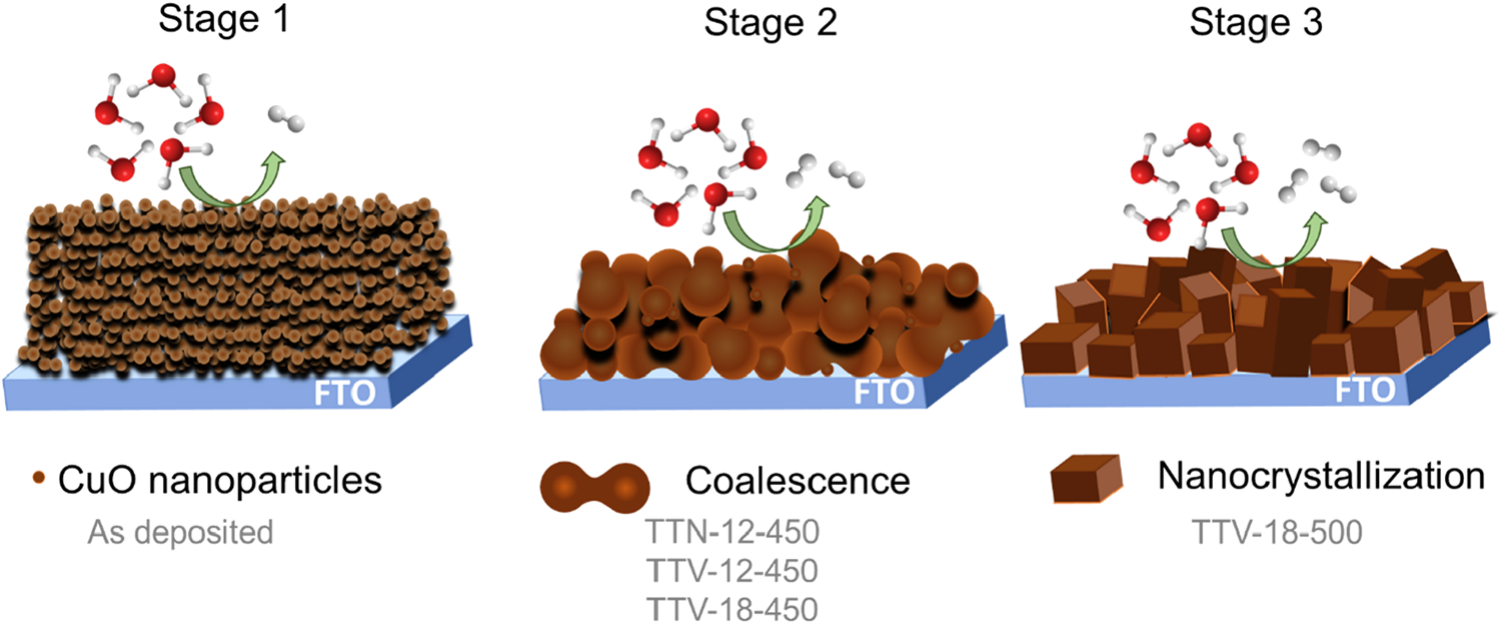
Scheme of the 3 Stages Reported on Nanocrystallite CuO Film Formation with Their Respective PEC Performance

Therefore, this work opens the route for the use of this gas-phase synthesis technique combined with thermal treatments, for the fabrication of nanostructured electrodes, not only of CuO, as we presented in this work, but also of any other oxide of elements that can be sputtered. As an example, the formation of nanostructured films from gas-phase deposited NPs has been previously reported for metallic V and Nb NPs, which were thermally treated in air to produce V_2_O_5_ and Nb_2_O_5_ nanostructures.^35,73^ Those investigations started from metallic NPs, and oxidation occurred during the thermal treatments, while the present study started directly from oxide NPs. Nevertheless, a notable similarity was observed in the morphological evolution as a function of the treatment temperature. In both cases, the individual as-deposited NPs presented a gradual coalescence with temperature (V_2_O_5_: 300–400 °C; Nb_2_O_5_: 500 °C) and subsequently evolved toward the formation of crystallites hundreds of nanometers in size at higher temperatures (V_2_O_5_: 500 °C; Nb_2_O_5_: 800 °C). The different temperatures at which the various morphologies appeared can be attributed to the distinct melting points of CuO, V_2_O_5_, and Nb_2_O_5_. In our case, the maximum treatment temperature was limited to 500 °C in order to preserve the integrity of the FTO electrode, although some structures of 200 nm could be observed in the SEM image (Figure 2c). Concerning the applicability of this methodology, it is also possible to apply this fabrication route to many other nanostructured oxides. This includes tuning the stoichiometry to obtain Cu_2_O electrodes,^74^ as well as modifying the composition to produce mixed oxides incorporating, for instance, two different transition metals. This approximation can be carried out either by starting from a sputtering target containing both metals or by taking advantage of the capabilities of the MICS system. Owing to the presence of three magnetrons in its aggregation zone, it is possible to fabricate nanoparticles combining different metals^75^ and to control their oxidation during the fabrication process.^34,76^ Therefore, it would be possible, for instance, to combine copper oxides with iron oxides to fabricate alternative CuFeO_2_ nanostructured electrodes^77,78^ or to incorporate tungsten oxide to improve photostability,^79^ as both Fe and W oxides have already been synthesized using this methodology.^80,81^

From the PEC results obtained using these CuO electrodes, we found a clear correlation between the crystallite size obtained after each thermal treatment and the photocurrent density generated, reaching the best performance for the sample grown after the TTV-18–500 treatment. Table S2 in Section S10 presents a summary of the results obtained in this work as a function of the thermal treatment. In our work, the evolution of morphology induced by the different thermal treatments plays a central role in determining the PEC response. Figure S17 shows the increased PEC performance, in terms of measured photocurrent, with increasing crystallite size. The as-deposited films, composed of randomly oriented CuO nanoparticles of 8 nm diameter, exhibit a highly nanogranular structure with a large number of interfaces, grain boundaries, and structural defects. These features hinder efficient charge transport across the film and promote electron–hole recombination, resulting in negligible photocurrent. Upon thermal treatment, the morphology progressively evolves through nanoparticle growth and coalescence processes. The initial stages (TTN and mild TTV conditions) lead to partial sintering and the formation of larger interconnected domains. This reduces the density of grain boundaries and improves charge percolation pathways, which explains the observed increase in photocurrent. More importantly, under optimized vacuum thermal treatment (TTV-18–500), a complete recrystallization of the sample occurs, leading to the formation of well-defined CuO nanocubes (∼200 nm) and a highly porous structure. This morphology provides several key advantages for PEC performance: (i) improved crystallinity and reduced defect density, as confirmed by XRD, which minimizes bulk recombination losses; (ii) enhanced charge transport, due to the formation of larger and better-connected crystalline domains; (iii) increased accessible surface area and open porosity, facilitating electrolyte penetration and interfacial charge transfer; and (iv) modified surface chemistry, as suggested by XPS, where the presence of oxygen-related defects may also contribute to improved catalytic activity.

The maximum photocurrent obtained is comparable to those reported for other CuO-based electrodes in the literature. For instance, this value is 3.4 times higher than that obtained for a Cu_2_O-CuO thin film heterostructure prepared by a sol–gel method, which reported a photocurrent of −0.35 mA· cm^–2^ at 0.05 V­(RHE).^82^ It also outperforms a CuO thin film fabricated by electrodeposition, which presented a photocurrent of −0.1 mA· cm^–2^ at −0.2 V vs Ag/AgCl.^83^ This highlights a promising avenue for future research, focused on developing heterostructures based on the CuO nanostructures presented in this work, aimed at improving photocurrent generation, charge separation, and extraction.

## Conclusions

In this study, nanostructured CuO films were synthesized on FTO substrates for their use as photocathodes. Starting from CuO NPs with an average diameter of 8 and 14 nm, as-deposited CuO NPs films with a 2.3 μm thickness evidenced a lack of photoelectrochemical activity, most probably related to the difficulty associated with achieving effective charge conduction across the CuO NP multilayer.

Thermal treatments of CuO NP films were needed to enhance their PEC performance. Heating the films at 450 °C for 12 h in a nitrogen atmosphere induced an Ostwald ripening process in the nanoparticles, resulting in initial NP growth followed by their coalescence. The treatment proved more effective when starting from smaller nanoparticles (8 nm diameter). At this stage, only low photocurrents were registered.

Thermal treatments in vacuum induced a more effective coalescence and led to larger grain sizes by increasing temperature (from 450 to 500 °C) and time (from 12 to 18 h). The treatment in vacuum for 18 h at 500 °C resulted in a complete recrystallization, with larger nanocrystalline domains in the form of nanocubes forming the film. This treatment presented the best PEC activity, with a photocurrent density of −1.2 mA·cm^–2^, a lower charge-transfer resistance and capacitance as well as an extended lifetime of the photogenerated electrons, as a consequence of improved charge generation, separation, and extraction. These results proved the importance of the structural and morphological properties of the material on the generated photocurrent. The larger grain sizes with better crystallinity and reduced defects lowered the recombination barriers, thereby optimizing the photogeneration of electron–hole pairs and enhancing charge transport. These findings highlight a promising strategy for the fabrication of CuO-based photoelectrodes by employing nanoparticles generated via a gas aggregation source, followed by thermal treatment to tailor the crystalline properties of the resulting layer. This approach paves the way for their application in photoelectrocatalysis, as the fabrication route can be applied to a wide variety of elements (virtually any material that can be sputtered) and their combinations to create more complex structures, such as mixed oxides.

## Supplementary Material



## Notes

Figure S1: AFM of the nanoparticles; Figure S2: FE-SEM of large CuO NPs as-deposited and after TTN; Figure S3: FE-SEM of small CuO NPs as-deposited and after TTN; Figure S4: FE-SEM of CuO NPs after TTVs; Figure S5: adsorption/desorption isotherms; Figure S6: EDX after TTV-18–500; Figure S7: XRD patterns; Table S1: calculated grain size; Figure S8. TEM images; Figure S9: Cu_LMM_ spectra; Figure S10: chronoamperometry and LSV after chronoamperometry; Figure S11: XRD after chronoamperometry; Figure S12: Cu 2p and O 1s XPS, and Cu_LMM_ spectra before and after the CA; Figure S13: calculated Tauc plots for an indirect optical transition; Figure S14: valence band XPS measurements; Figure S15: fits of photoluminescence decay; Figure S16: fits of transient decay; Table S2: summary of the fabrication parameters and the resulting data of the electrodes; and Figure S17: maximum current densities vs crystallite sizes (PDF)
