# Supplementary material for: Highly Controlled Nanostructured CuO Photocathodes
Source: ACS Appl Nano Mater. 2026 Jun 27;9(27):12748–62. doi: 10.1021/acsanm.6c01037 (PMC13366653; doi:10.1021/acsanm.6c01037)
Supplement: Supplementary file 1 [file an6c01037_si_001.pdf]

Supporting Information

Highly Controlled Nanostructured CuO Photocathodes

Javier Prieto-Serrano[1], Miguel García-Tecedor[2], Mariam Barawi[2], Miguel Gomez-Mendoza[2], María Alcaire[3], Ana Borras[3], Víctor A. de la Peña O'Shea[2], José A. Martín-Gago[1], María F. López[1]*‡, Lidia Martínez[1]*‡*

[1] Instituto de Ciencia de Materiales de Madrid (ICMM), CSIC, Madrid, 28049, Spain.

[2] IMDEA-Energía, Móstoles, 28935, Spain.

[3] Instituto de Ciencia de Materiales de Sevilla (ICMS), CSIC – US, Sevilla, 41092, Spain.

**Corresponding authors: M. García-Tecedor (miguel.tecedor@imdea.org), M.F. López (mflopez@icmm.csic.es); L. Martínez (lidia.martinez@icmm.csic.es)*

S1. Morphological characterization of the fabricated electrodes

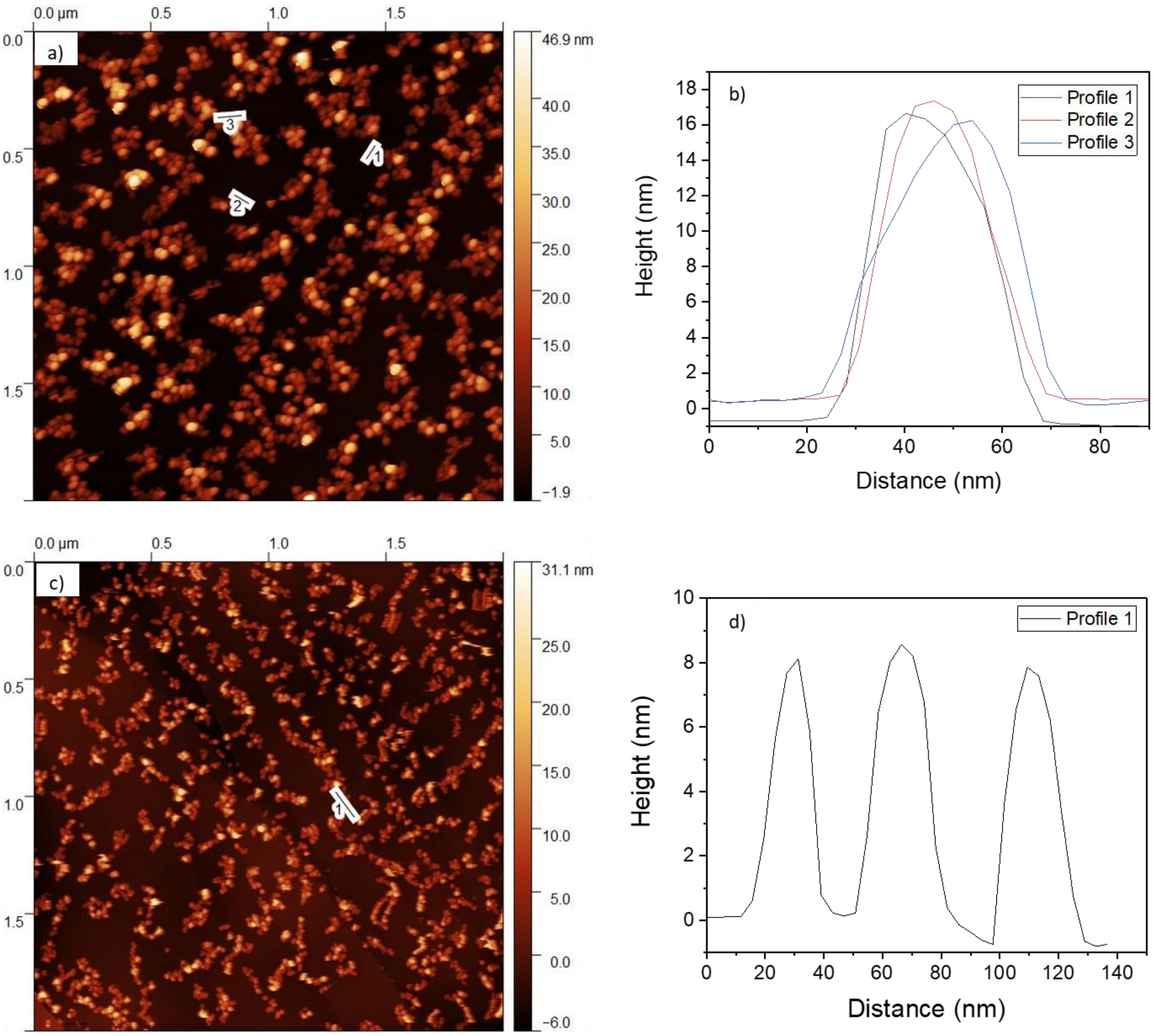

Figure S1. (a) Representative AFM image of (a) large and (c) small CuO NPs deposited on HOPG. (b) and (d) height profiles of white lines depicted in (a) and (c), respectively. The NP size was calculated by measuring their height in order to avoid the tip-sample convolution [1] .

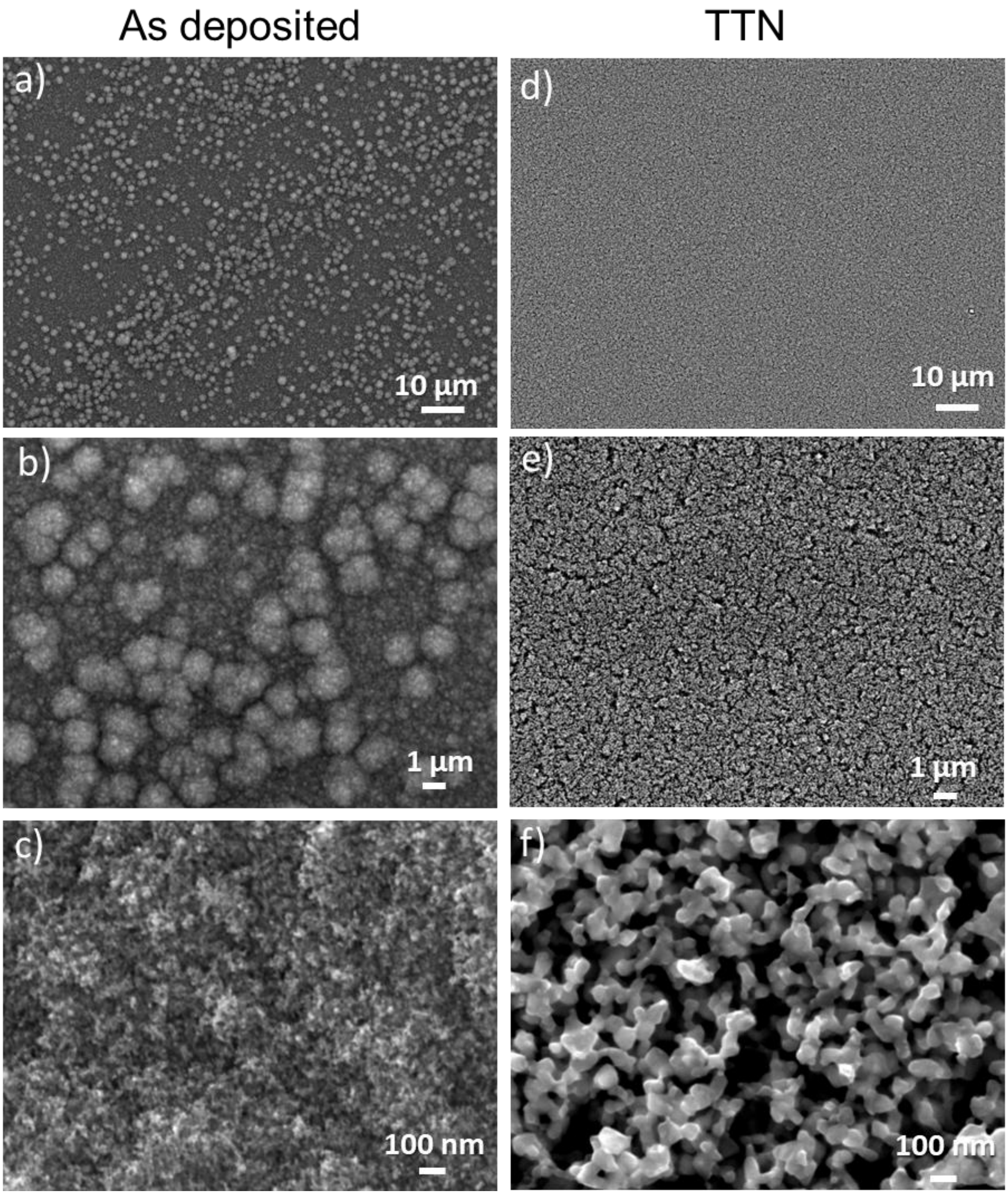

Figure S2. FE-SEM of large CuO NPs on FTO (left) as deposited and (right) after TTN for 12h at 450ºC. Top view images at different magnifications.

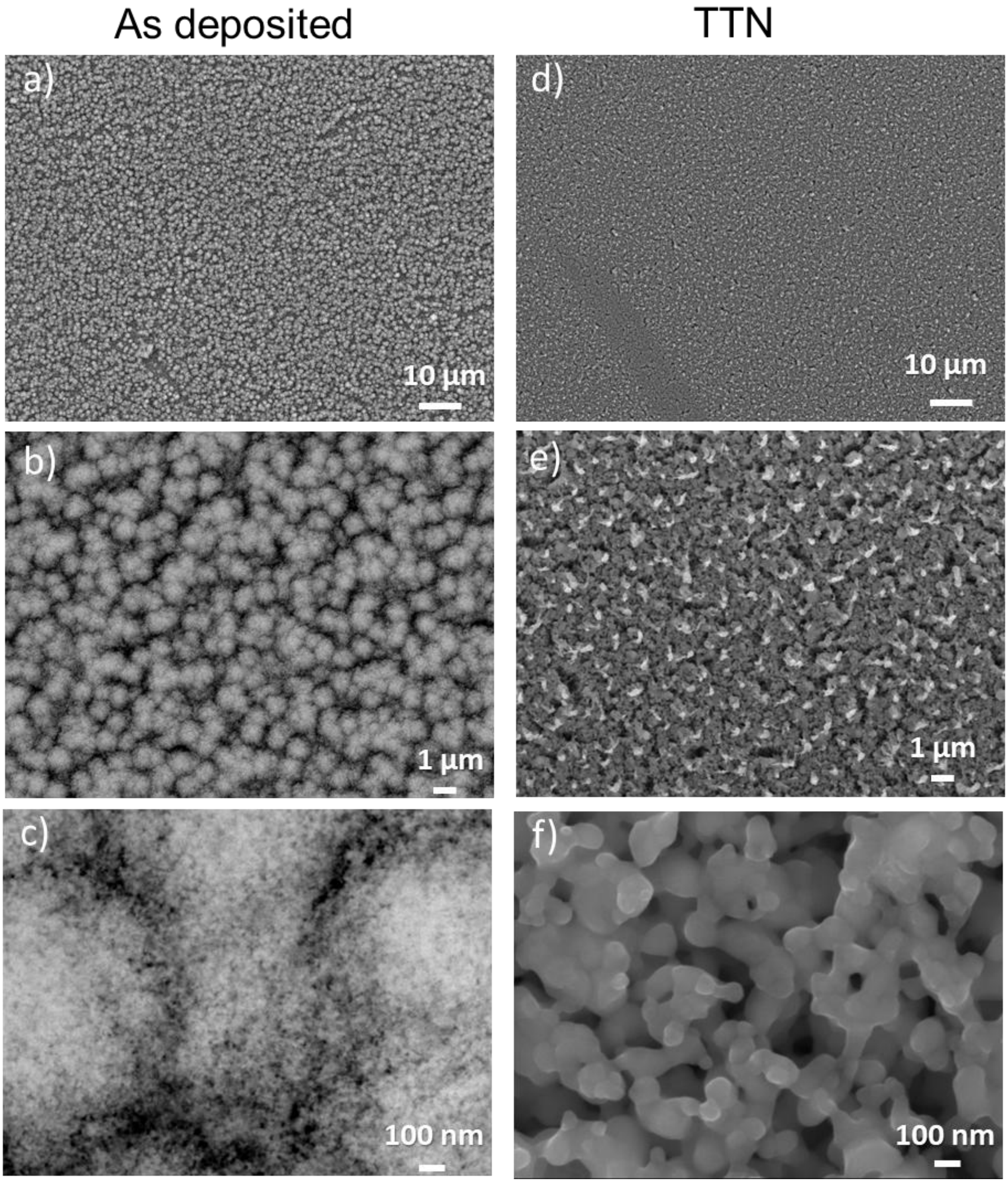

Figure S3. FE-SEM of small CuO NPs on FTO (left) as deposited and (right) after TTN for 12h at 450ºC. Top view images at different magnifications.

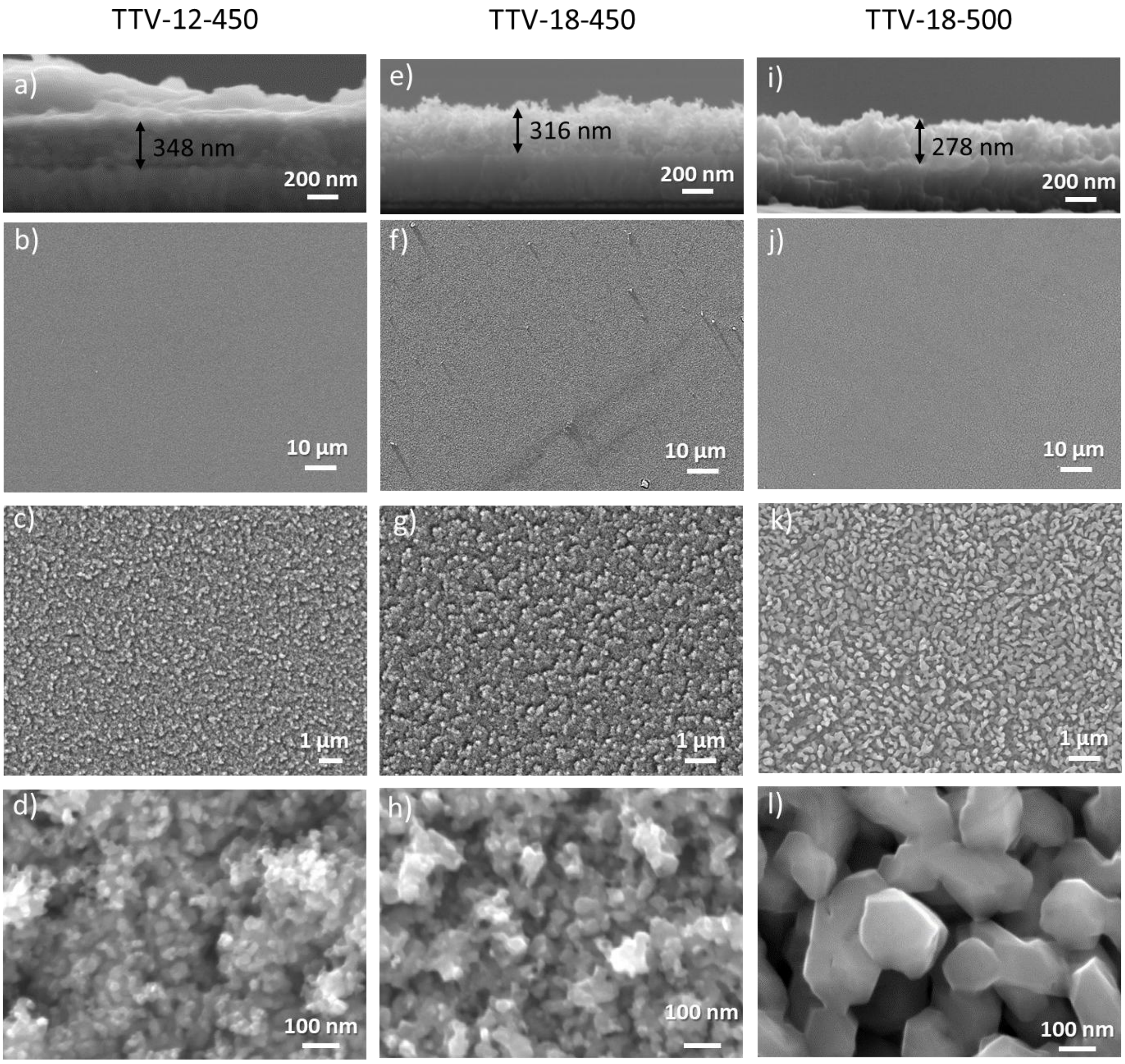

Figure S4. FE-SEM images at different magnifications of the FTO/CuO electrodes fabricated with small CuO NPs. (a-d) TTV- 12-450, (e-h) TTV-18-450, (i-l) TTV-18-500. Cross sections (top) and top view images at different magnifications.

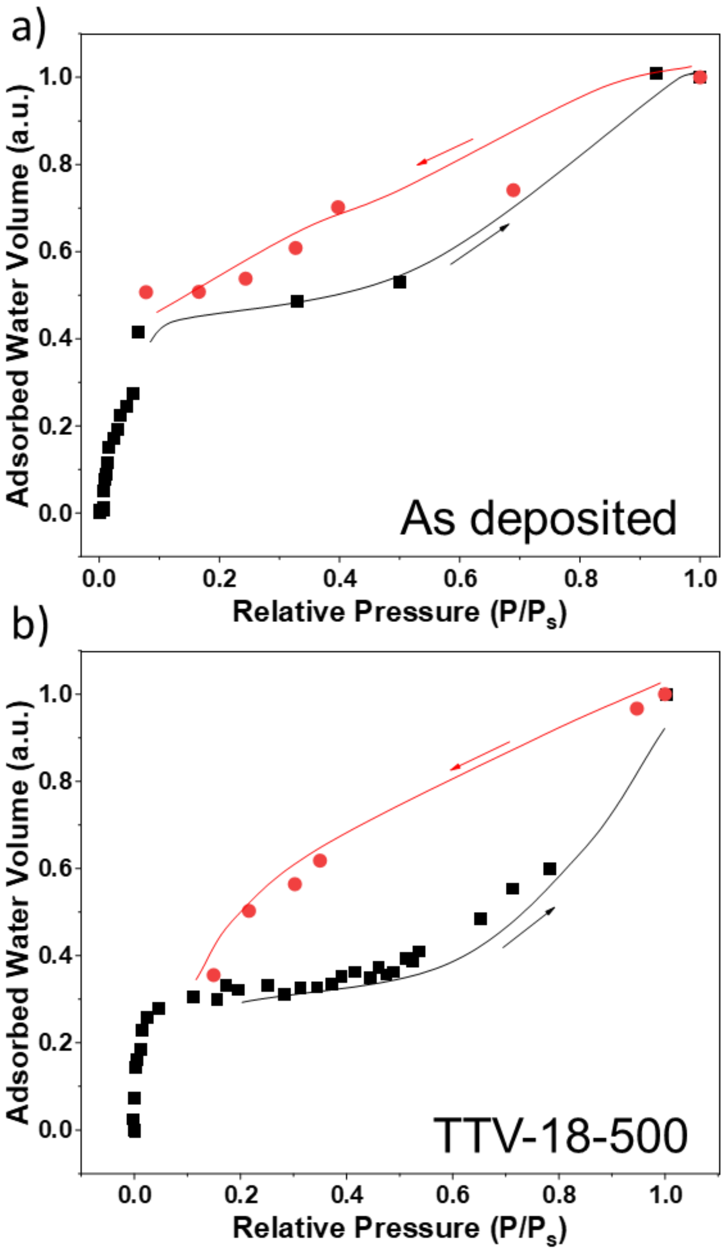

Figure S5. Normalized adsorption/desorption isotherms (19.5 ºC) of water on as-deposited and TTN NPs acquired by the QCM method. The arrows indicate the adsorption (pointing right) and desorption (left) branches of the isotherms.

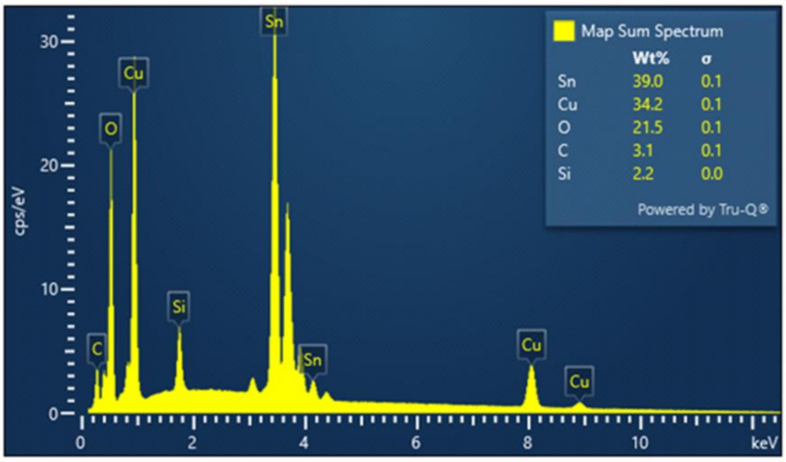

Figure S6. EDX spectrum of TTV-18-500 sample.

S2. X-ray diffraction of the fabricated electrodes

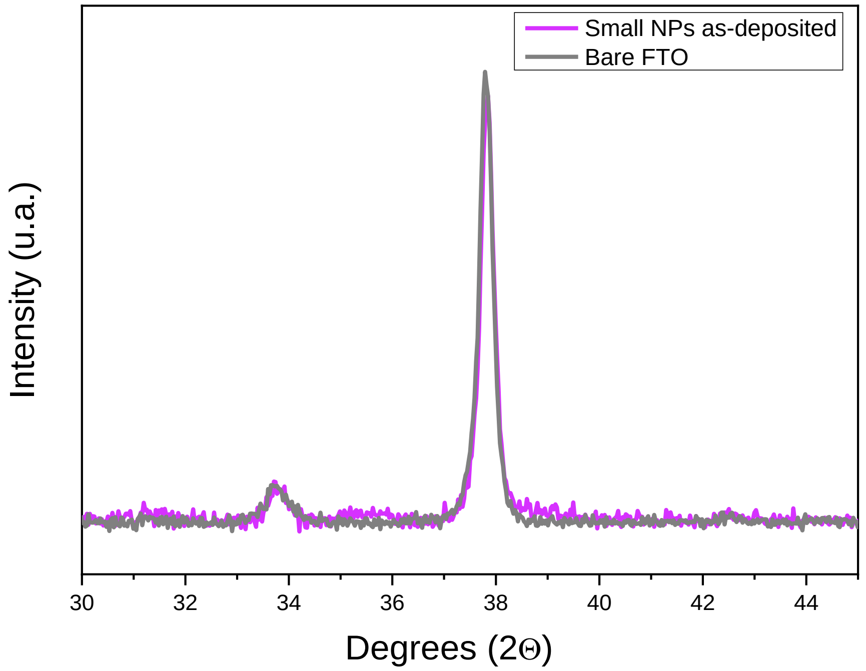

Figure S7. XRD patterns of as-deposited multilayer of NPs and bare FTO.

Table S1. Calculated values of the mean crystallite sizes obtained using the Debye Scherrer equation for the CuO peaks at 36.6º and 38.8º from the XRD diffractograms of figure 2g.

	**Mean crystallite size (nm)**		
	**TTV-12-450**	**TTV-18-450**	**TTV-18-500**
**Plane of peak (111)**	23	30	45
**Plane of peak (002)**	15	19	34

S3. Transmission Electron Microscopy (TEM)

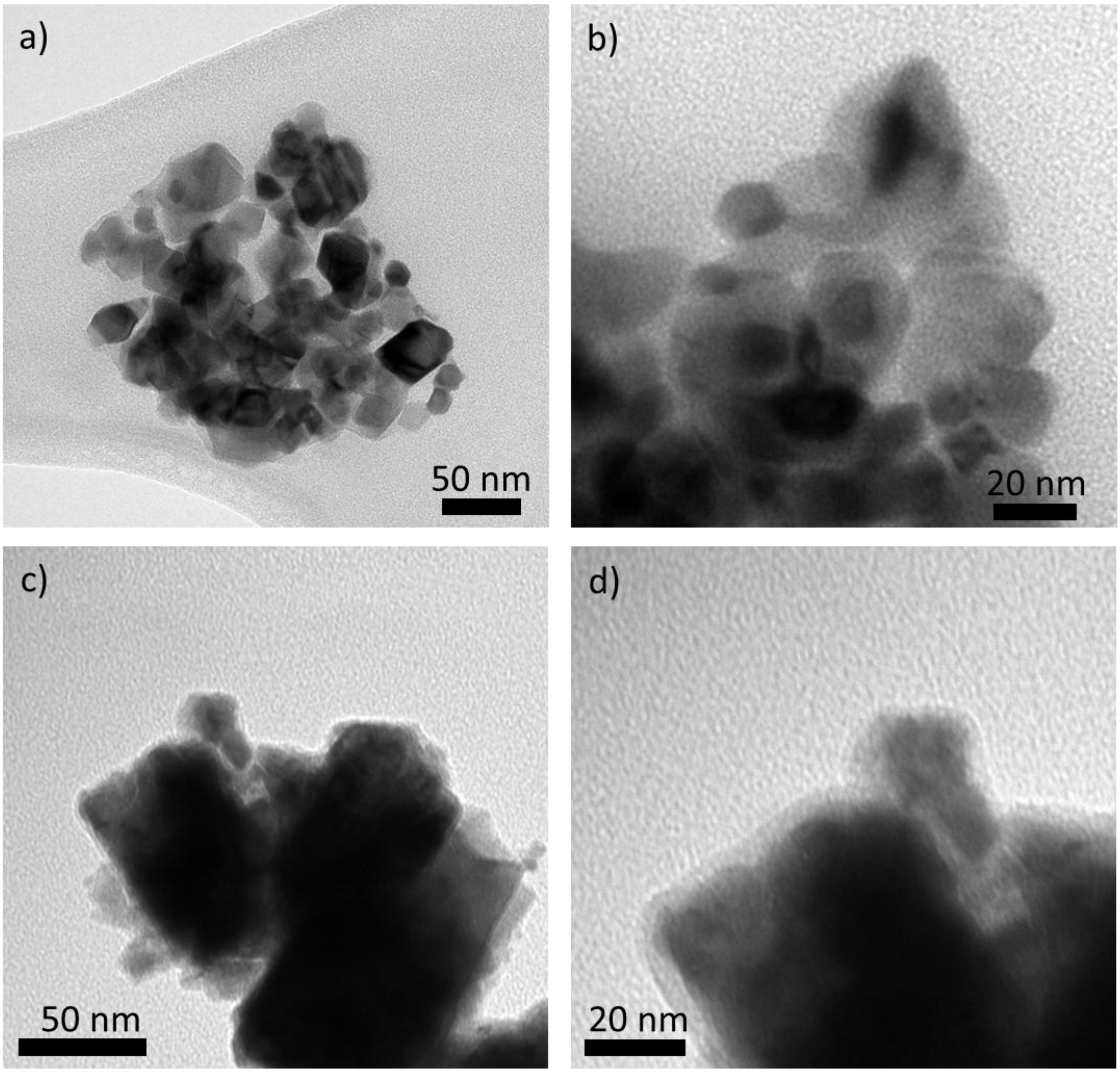

Figure S8. TEM images of the (a, b) TTV-12-450 and (c, d) TTV-18-500 samples, respectively.

S4. XPS of the fabricated electrodes

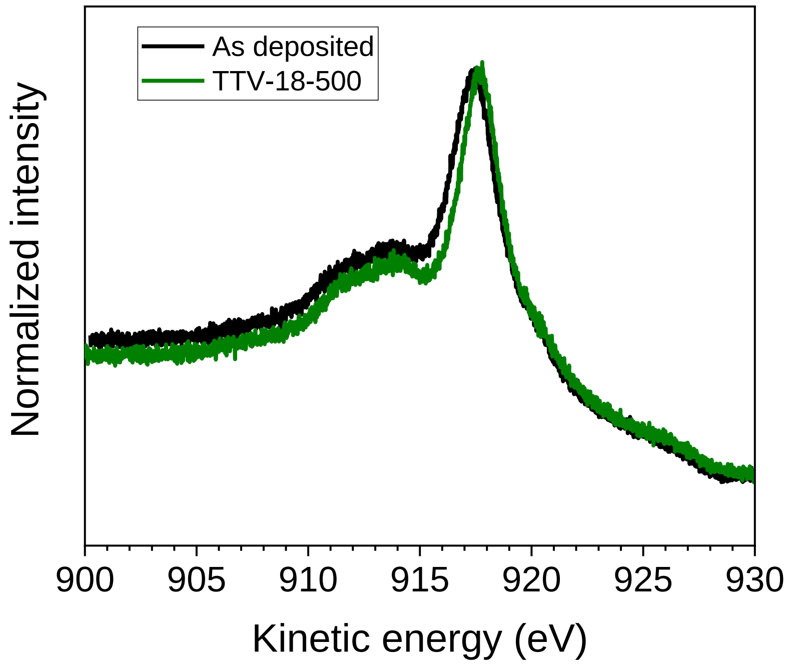

Figure S9. Cu_{LMM} spectra of as-deposited NPs (black) and after TTV-18-500 (green).

S5. PEC stability

After CA, a LSV was performed to TTV-18-500 electrode (see **Figure S10**). An electrochemical peak between 0.0 V and 0.2 V vs RHE was observed, which confirms a loss of activity and degradation.

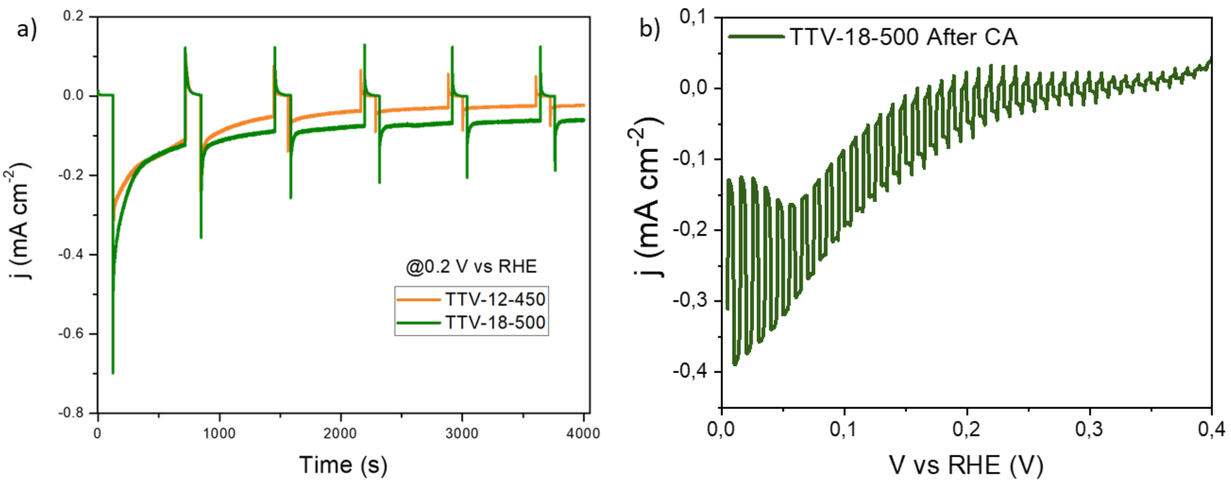

Figure S10. a) Chronoamperometry (CA) of TTV-12-450 (orange) and TTV-18-500 (green) electrodes at a fixed potential of 0.2V vs RHE, starting in darkness condition (2 min) and under light (10min) in 5 cycles. b) LSV of TTV-18-500 electrode after CA.

S6. Chemical stability

In order to understand the cause of dropping in activity of the electrode after CA, it was analysed by XRD and XPS. **Figure S11** presents a comparison of the XRD before (dark green curve) and after CA (light green curve). Before CA, only CuO [2] peaks were observed, while in After CA, Cu_2O [3] peaks were also registered. It should be noted that the Cu_2O [3] peaks are of greater intensity than the CuO [2] peaks, which indicates an almost complete electrochemical reduction of the material, which is consistent with the performance of the electrode in **Figure S10**.

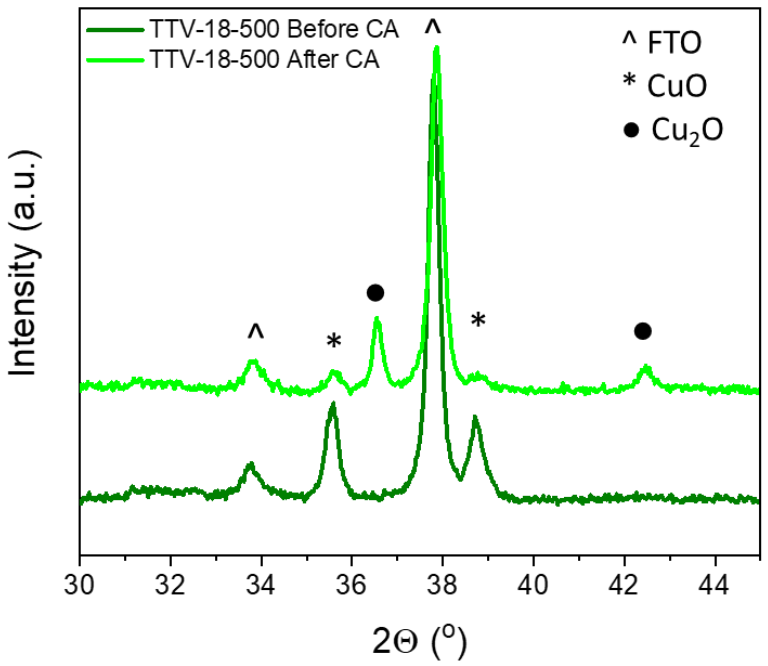

Figure S11. XRD patterns of TTV-18-500 electrode before and after the CA.

Figure S12 displays the XPS measurements after PEC stability tests compared to the ones showed in **Figure 2 h,i.** It can be clearly observed a partial reduction to Cu^+, having mostly Cu^+ emissions, with also contributions from the original CuO.

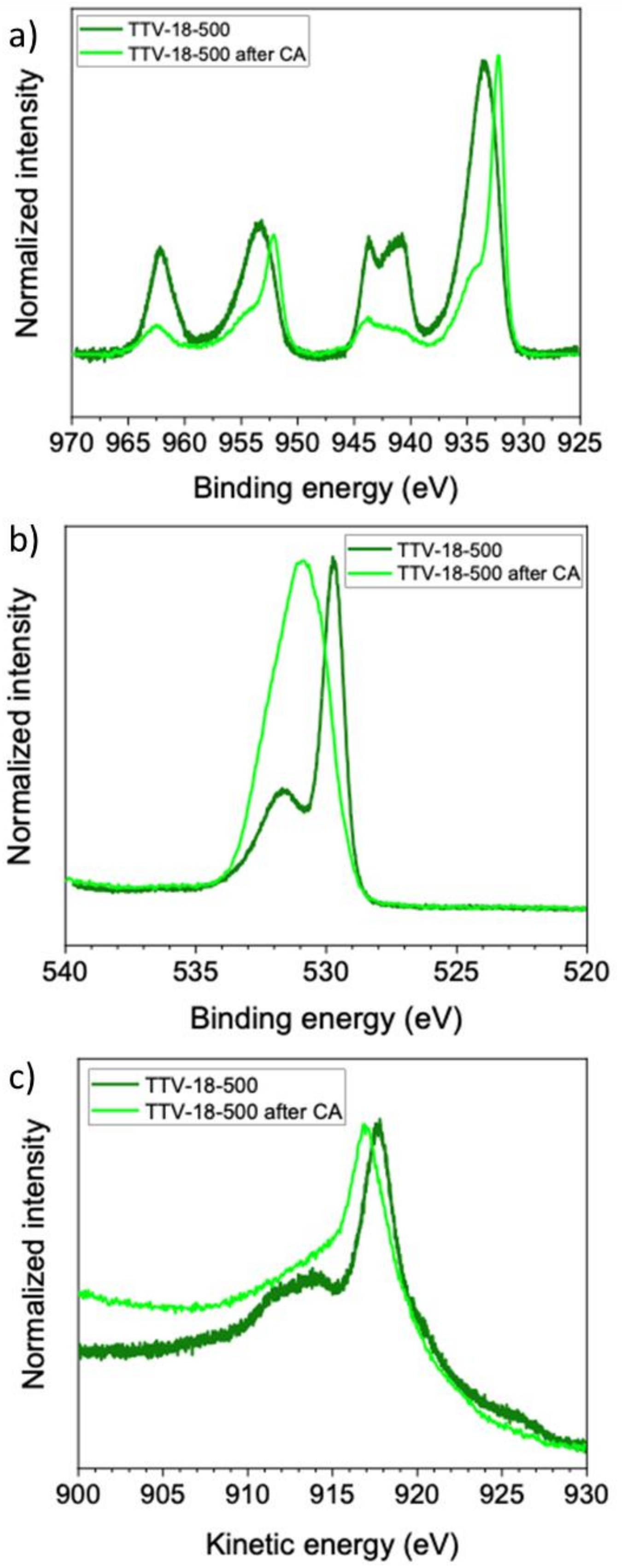

Figure S12. a) Cu 2p, b) O 1s and c) Cu_{LMM} spectra of TTV-18-500 electrode before and after the CA.

S7. Optical characterization

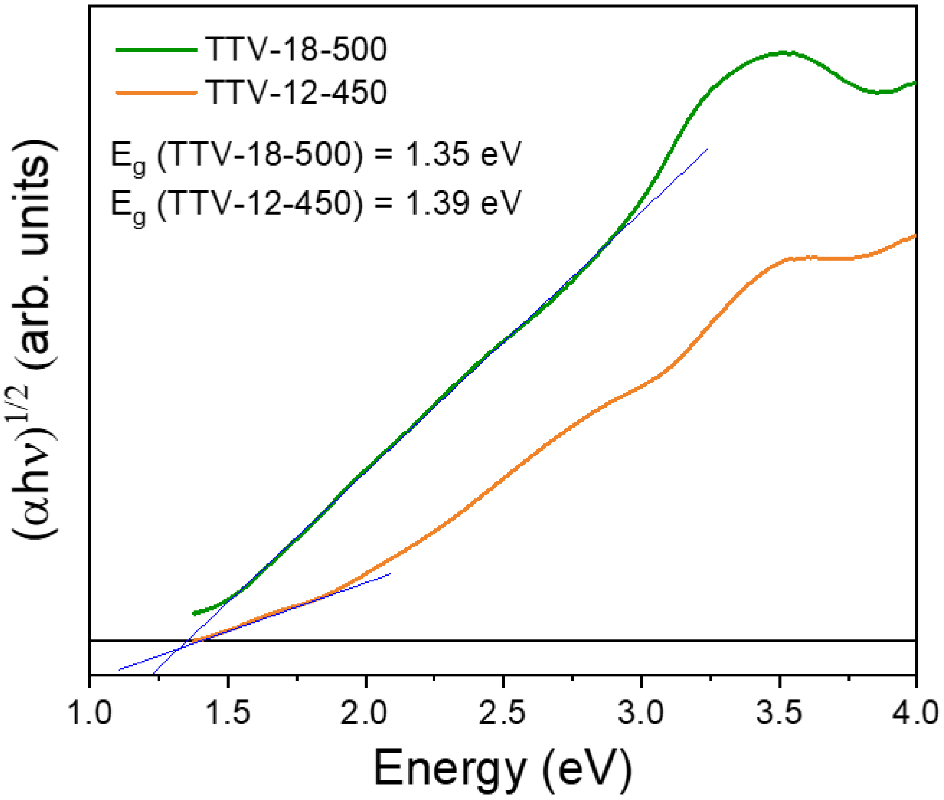

Figure S13. Calculated Tauc plots for an indirect optical transition in the fabricated CuO photocathodes.

S8. Valence band measurements

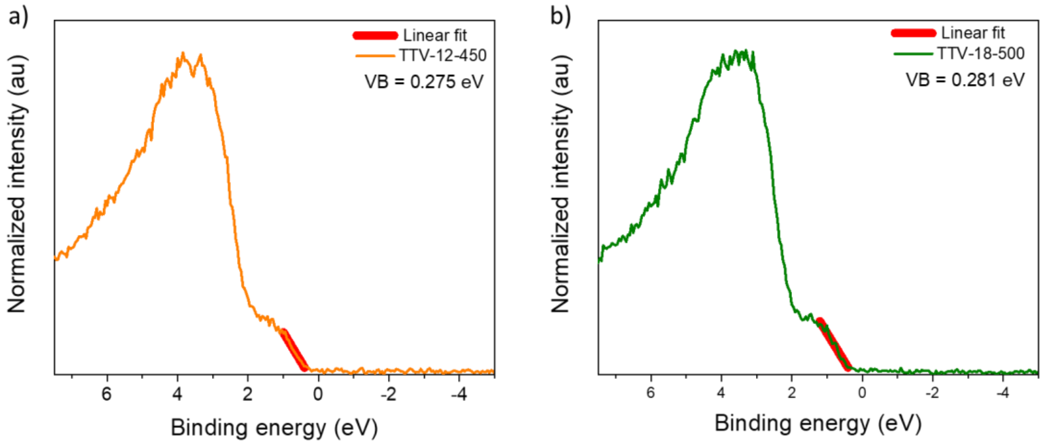

Figure S14. Valence band XPS measurements of the a) TTV-12-450 and b) TTV-18-500 samples.

S9. Photoluminescence tests.

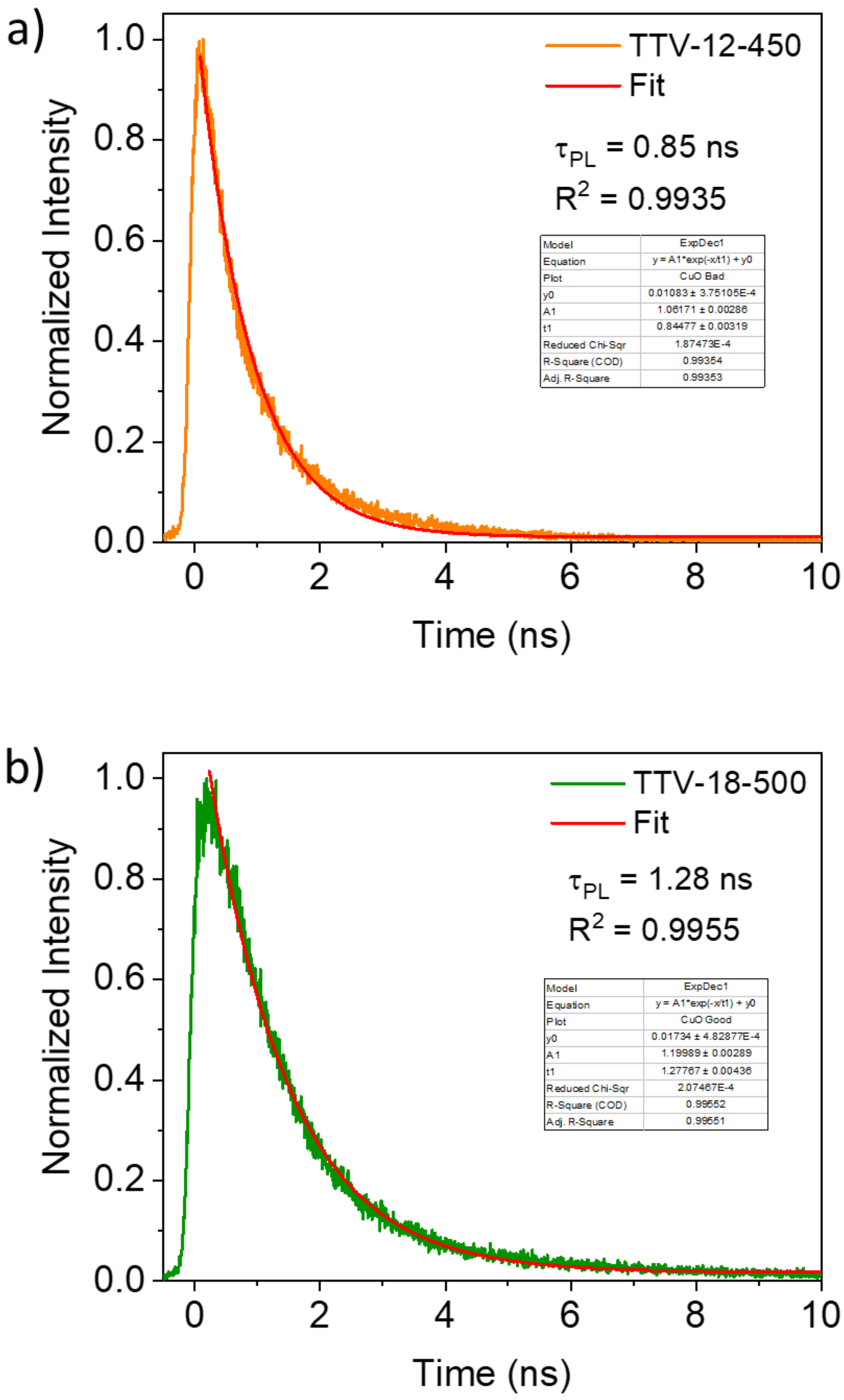

Figure S15. Fits of the photoluminescence decay traces (λ_{exc} = 372 nm, band-pass filter between 475-525 nm) for a) TTV-12-450 CuO electrode (orange) and b) TTV-18-500 CuO electrode (green).

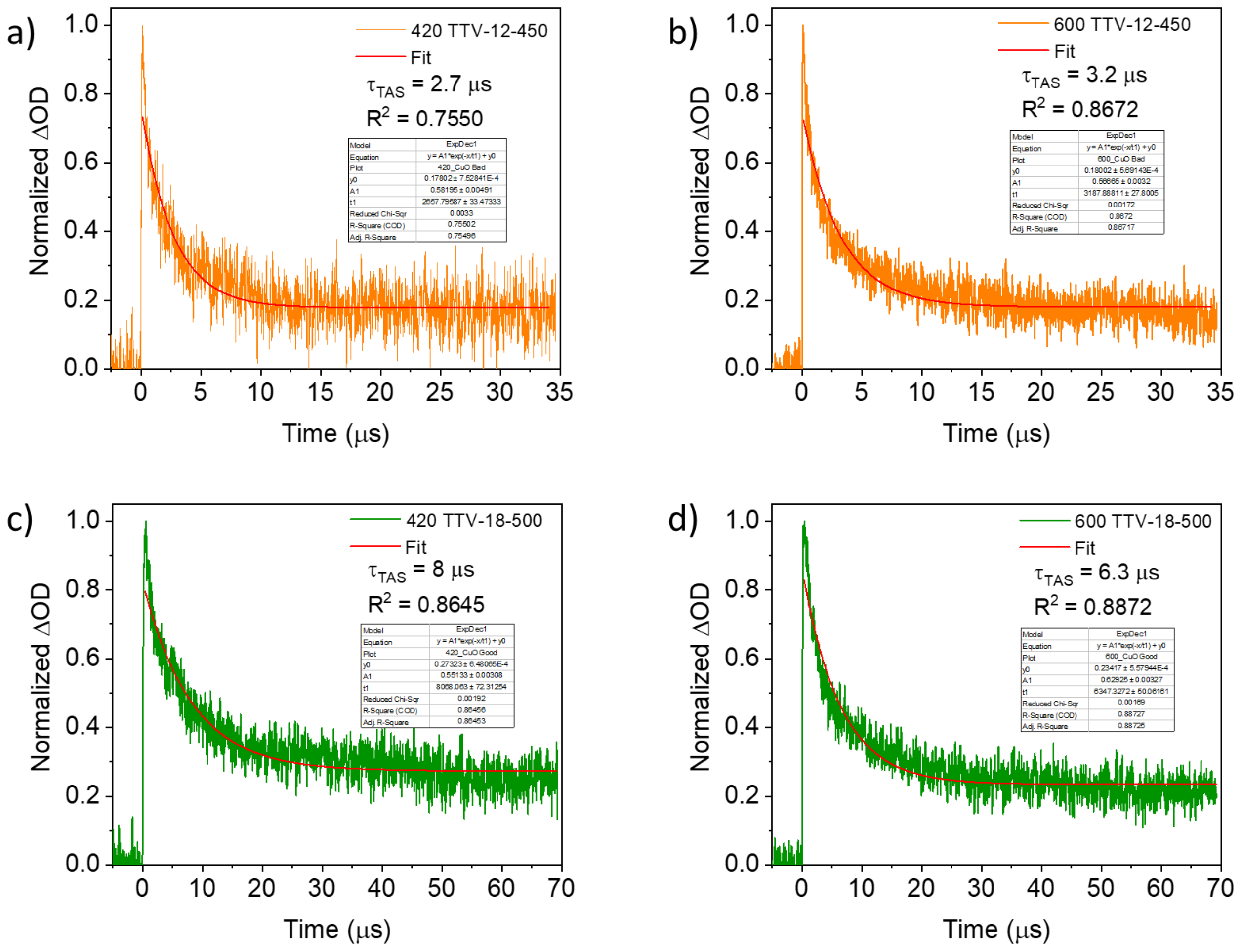

Figure S16. Fits of the transient decay traces (λ_{exc} = 355 nm, λ_{obs} = 420 or 600 nm) for the TTV-12-450 (orange) and TTV-18-500 (green) CuO-based electrodes.

S10. Summary of the results obtained of the electrodes fabricated.

Table S2 summarizes the characterization of the electrocathodes fabricated after each TTV. The values displayed are extracted from the XRD (preferential planes and crystallite size), AFM (NP size), SEM (nanostructure size) and LSV (current density) presented in the manuscript. These data clearly indicate the best performance of TTV-18-500, where even an applied bias photon-to-current efficiency of 1.4 % was obtained.

Table S2. Summary of the fabrication parameters and the resulting data of each type of electrode extracted from the XRD, AFM (as-deposited)/SEM (TTV) and LSV.

Sample	Thermal treatment in vacuum	Preferential planes	Crystallite size (nm)	Nanostructure size (nm)	j_{max} (mA/cm^2)
As-deposited	-	Random	-	8	-0.01
TTV-12-450	12 h / 450 ºC	(111), (002)	23	20 - 100	-0.5
TTV-18-450	18 h / 450 ºC	(111), (002)	30	50 - 100	-0.75
TTV-18-500	18 h / 500 ºC	(111), (002)	45	200 - 500	-1.2

From these data it is clear the direct correlation between increasing time and temperature of the TTVs with the increasing crystallite and nanostructure size, that consequently leaded to the generation of larger photocurrents. In particular, if we focused on the evolution of the photocurrent generated vs. the crystallite size measured by XRD, we found a linear relation between both parameters according to y = 0.24 (± 0.02) - 0.0322 (± 0.0007) x (Figure S13). It has to be taken into account that this line also passes through the point corresponding to the as-deposited NPs, considering 8 nm in size extracted from the AFM (cannot be extracted from XRD) and the -0.01mA/cm2 obtained with those electrodes (see hollow circle in Figure S13).

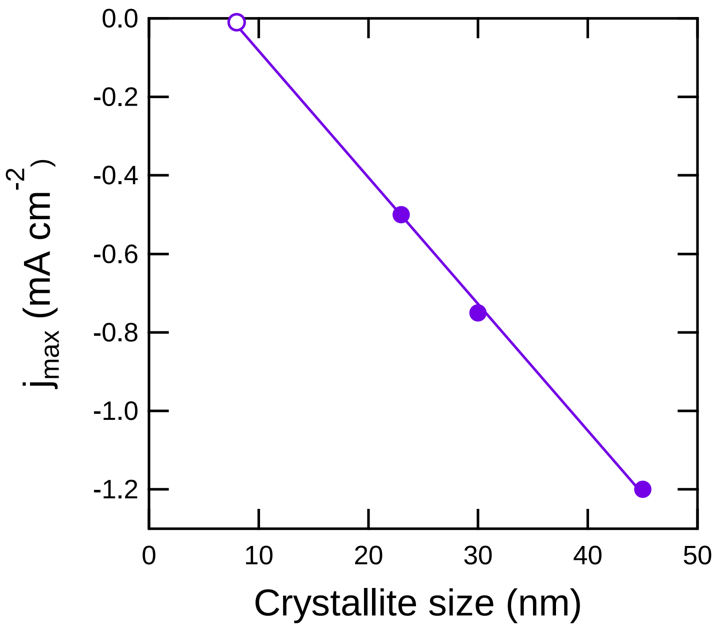

Figure S17. Evolution of the maximum current density measured in the LSV vs. crystallite size measured by XRD.

REFERENCES

(1) Martínez, L.; Tello, M.; Díaz, M.; Román, E.; Garcia, R.; Huttel, Y. Aspect-Ratio and Lateral-Resolution Enhancement in Force Microscopy by Attaching Nanoclusters Generated by an Ion Cluster Source at the End of a Silicon Tip. *Review of Scientific Instruments* **2011**, *82* (2). https://doi.org/10.1063/1.3556788.

(2) PDF Card - 00-045-0937.

(3) PDF Card - 01-071-4310.
